# Time-Dependent Contact Behaviour of ZDDP-Derived Tribofilms: A Viscoelastic Layered Model Approach

**DOI:** 10.1007/s11249-025-01990-5

**Published:** 2025-05-03

**Authors:** Dongze Wang, Ali Ghanbarzadeh, Nan Xu, Qingyang Liu, Gregory de Boer

**Affiliations:** 1 School of Mechanical Engineering, University of Leeds, Leeds, LS2 9JT UK; 2 Qatar Environment and Energy Research Institute, Doha, Qatar

**Keywords:** Contact mechanics, ZDDP, Viscoelasticity, Layered contact

## Abstract

The ZDDP-derived tribofilm was recently reported to be viscoelastic based on a creep experiment, where a Burgers material model mathematically represents its creep compliance. This study develops a contact model for layered materials by extending a previously established viscoelastic half-space contact model. The approach involves converting analytical frequency response functions into influence coefficients, enabling the investigation of the viscoelastic behaviour of ZDDP-derived tribofilms. The results reveal that the tribofilm exhibits a highly fluid-like response when modelled as a half-space body being in contact with a carbon steel ball during indentation or sliding. When bonded to an elastic substrate in its typical thin-film form (on the nanometre scale), the contact behaviour can still exhibit time-dependent characteristics, depending on the operating conditions. Creep and stress relaxation are observed during indentation, particularly under low loads, while high loads result in a more pronounced viscoelastic response in extremely slow-speed contacts. However, under moderate sliding speeds ranging from millimetres to meters per second, time-dependent effects become negligible, regardless of the applied load. These findings indicate that although ZDDP-derived tribofilms exhibit significant viscoelasticity, their behaviour in practical applications generally resembles that of a soft elastic layer, as typical sliding speeds fall outside the range where pronounced time-dependent effects occur.

**Graphical Abstract:**

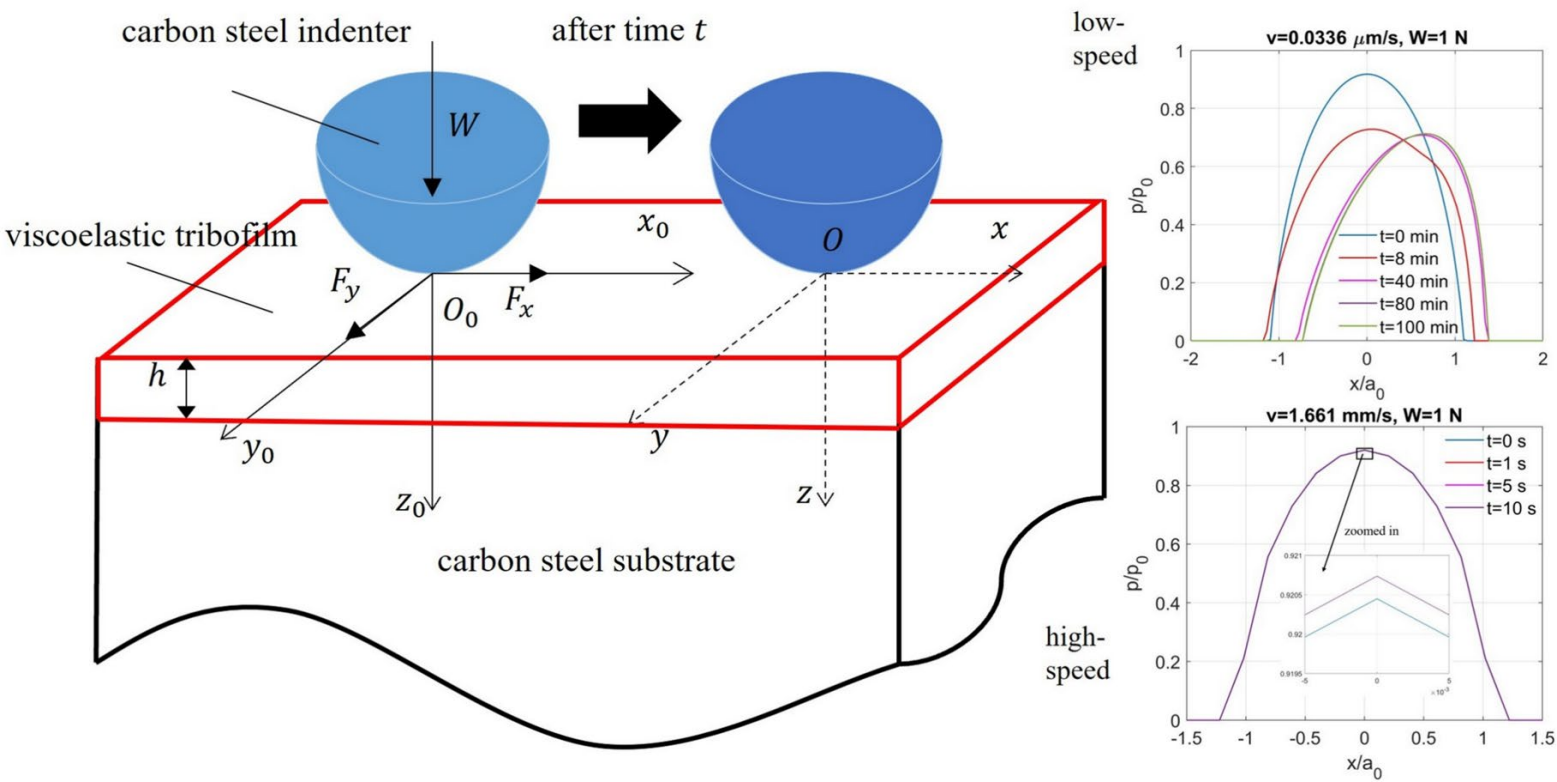

**Supplementary Information:**

The online version contains supplementary material available at 10.1007/s11249-025-01990-5.

## Introduction

Zinc dialkyl dithiophosphate (ZDDP) is one of the most common anti-wear additives applied in various industries considering its outstanding ability to generate protective films known as ZDDP-derived tribofilms on contacting surfaces [1, 2]. In addition to its anti-wear properties, ZDDP works as a multifunctional additive providing both anti-oxidant and anti-corrosion performance. Although its usage brings certain benefits to the system, ZDDP presents several drawbacks. The generated tribofilms have been argued to aggravate the micro pitting in rolling contacts due to the consequent high friction and low wear rate [3, 4]. The running-in of rough surfaces was found to be hindered due to this protective tribofilm, which leads to plastic deformations and stress concentrations at surface asperities. This intensifies subsurface stress fields, increases the likelihood of micro-crack formation and propagation, and elevates the risk of surface fatigue. The severity degree of micro pitting increases with the thickness of tribofilm, which leads to the reduction of bearing service life [5]. In addition, due to the presence of sulphur and phosphorus, when ZDDP is applied to systems equipped with catalytic converters such as the exhaust system of vehicles, it can poison the catalyst producing harmful emissions [6, 7], although its performance is highly dependent on the two chemical elements [8]. Furthermore, ZDDP additives have been argued to be unsuitable for electrical vehicle (EV) and hybrid transmission systems [9]. While they provide anti-wear benefits, their sulphur content can corrode copper components, thus undermining motor functionality [10]. Additionally, ZDDP-derived tribofilms can increase electric resistance at contact points [9], leading to local overheating, oxidation, and wear under bearing currents [11, 12]. More recent studies showed that while ZDDP continues to provide wear protection in electrified environments [13], the electric currents hinder the formation of optimal protective films, which can compromise the anti-wear performance under certain conditions [13, 14]. To address these issues, e-fluids tailored for EVs are being developed to meet lubrication needs without sacrificing component integrity [15, 16]. With EVs gaining popularity and stricter limits on ZDDP usage, it is crucial to develop sulphur-free and eco-friendly alternatives with comparable performance. To completely replace ZDDP, knowledge about ZDDP-derived tribofilms in terms of their mechanical properties, kinetics, morphology, and relevant rheological study is necessary.

Although ZDDP-derived tribofilms have been regarded as a solid-like surface film that reacts elastically under normal loads for a long time [17], the viscoelasticity of tribofilms has been developed from a proposed assumption to an experimental finding. It was first proposed by Heinike [18] that the film inside the tribological contact should be in a magma state (an extremely viscous high-temperature liquid) and likely in the plasma state (a high-temperature ionized gas). A breakthrough was achieved by Pidduck and Smith [19], who found the existence of a soft and viscous overlayer covering the solid-like surface film. The presence of the viscous layer was further proved by the relevant chemical analysis on tribofilms [2, 20, 21]. From the pin-on-plate test by Minfray, et al. [22], the pads of tribofilms were found to elongate in the direction of sliding implying that the flow of the tribofilm is similar to a fluid. Numerical results of their developed molecular dynamic model [22] showed a particular rheological behaviour of the zinc polyphosphate under friction conditions suggesting a zinc phosphate layer modification from a solid-like to a liquid-like phase under extreme conditions. Besides, the compositional gradient within the tribofilm [8] was argued to induce varying rheological behaviours across the tribofilm thickness such that the layers of tribofilms close to the metal surface perform most likely elastic [23, 24] while the out layers are more viscous [19, 25, 26]. Dorgham [27] recently reported that ZDDP-derived tribofilms behave as viscoelastic interfaces between rubbing surfaces, exhibiting fluid-like flow and spreading under shear. Through creep and squeeze-flow experiments, Dorgham, et al. [25] quantified the viscoelastic properties of these tribofilms. They found that the high viscosity of tribofilms can contribute to their superior anti-wear performance by allowing them to flow during formation (under shear) and to maintain nanoscale organization through adaptive movement of tribofilm pads at the contact interface. Notably, this extremely fluid-like state of the tribofilms was argued to hold during the period of rapid film formation [26] since the initially amorphous structure of tribofilms tends to become crystalline under continued rubbing.

In the meantime, most solutions within the framework of classical contact mechanics [28, 29] are heavily dependent on the half-space approximation, where the thickness of contacting solids is considerably larger than the contact radius. However, owing to the nature of viscoelastic materials (e.g. low contact compliance) and the time-varying material creep, the viscoelastic contacting area is usually larger than the ordinary elastic solid. Indeed, a viscoelastic surface layer (e.g. the ZDDP-derived tribofilm investigated in the current study) has often to be accounted for many engineering surfaces. In these cases, the surface layer or coating always exhibits characteristics that are significantly different compared with the remaining bulk region of the contacting body. Therefore, the reliability of contact analysis based on the half-space approximation is undermined on this occasion. As argued by Pauk and Wozniak [30], Chen and Chen [31], and Zhang, et al. [32], viscoelastic materials such as polymers mainly exist as a finite film instead of a half-space when it comes to their application. The thin layers, or a more general layer-substrate system, are widely employed in engineering systems, such as gears with viscoelastic coatings, seals of finite thickness, and bearings with anti-wear layers. A model simulating the contact behaviour of these surface films is of fundamental importance in tribological applications, which shall be helpful for the analysis or design of relevant engineering components.

Over the past decades, great efforts have been made in solving the indentation, sliding, or rolling contact problems of viscoelastic layers. The first attempt was made by Batra and Ling [33], who investigated the surface deformation, friction dissipation, and contact stresses of a viscoelastic-elastic layered system under the influence of a moving load. Later, Naghieh, et al. [34, 35] proposed analytical solutions to the frictionless indentation problem of a rigid smooth indenter against a single-layered viscoelastic material bonded to a rigid substrate. The key roles played by the viscoelasticity and sliding velocity in the distributions of contact pressure and internal stress (e.g. non-symmetrical pressure profile caused by a viscoelastic layer) were reported by Goryacheva, et al. [36] in their study about the contact of a rough body against a viscoelastic layered semi-infinite plane. The different indentation problems between layers of arbitrary viscoelastic materials, including elliptical [37], rebound spherical [38] and cylindrical contact [39], were investigated analytically by Argatov and Mishuris. However, these proposed solutions are limited by the assumption of monotonic loading. A more general solution to indentation problems of viscoelastic layered materials was developed by Chen, et al. [40] using the Hankel transform. By extending Persson’s theory of contact mechanics to layered materials [41], Scaraggi and Persson [42] investigated the behaviour of a viscoelastic layer in rough surface contact. Effects of finite roughness size and rubber thickness were studied in their study.

Regarding the numerical attempts for layered contact of viscoelastic materials, a Boundary Element Method (BEM)-based model was developed by Carbone and Putignano [43, 44] to simulate steady-state sliding contact problems. A correction factor, which takes the thickness of the viscoelastic surface and sliding speed into account, was introduced in their novel formulation of Green’s function. The model was then extended to investigate the effect of the thickness of viscoelastic layer in rough surface contact [45, 46] and the problem of anisotropy induced by the viscoelasticity in rough sliding contact problems [47]. The two different material combinations, including a viscoelastic layer bonded with a rigid half-space and a viscoelastic half-space covered by a rigid surface layer, were simulated by Torskava and Stepanov [48]. The effects of the surface layer on the hysteretic losses during the viscoelastic sliding contact were reported in the study. A more general transient analysis of frictionless sliding viscoelastic problems was recently proposed by Wallace, et al. [49]. The involved layer and substrate can exhibit different properties (either elastic or viscoelastic). The problem of imperfect bonding between layer and substrate in viscoelastic sliding contact was analysed numerically by Zhang et al. [50]. To date, the framework of numerical modelling for viscoelastic layered contact has been initially established, but the application of these models to real-life contact scenarios is rather limited.

In this study, by developing the elastic layered contact model first based on the well-established theory of elastic contact, a BEM-based model providing transient contact analysis of viscoelastic layered surfaces was developed after converting the elastic layered contact model through the elastic–viscoelastic correspondence principle. A Burgers material model was built to characterise the contact behaviour of the ZDDP-derived tribofilm by fitting the four-term equation with the creep compliance curve reported by Dorgham, et al. [25]. By using the specific properties of the ZDDP-derived tribofilm as the material input for the layer, the role played by the viscoelasticity of the tribofilm under different contact conditions, including normal indentation and sliding, was exposed numerically for the first time.

## Theory and Algorithm Description

**Fig. 1:**
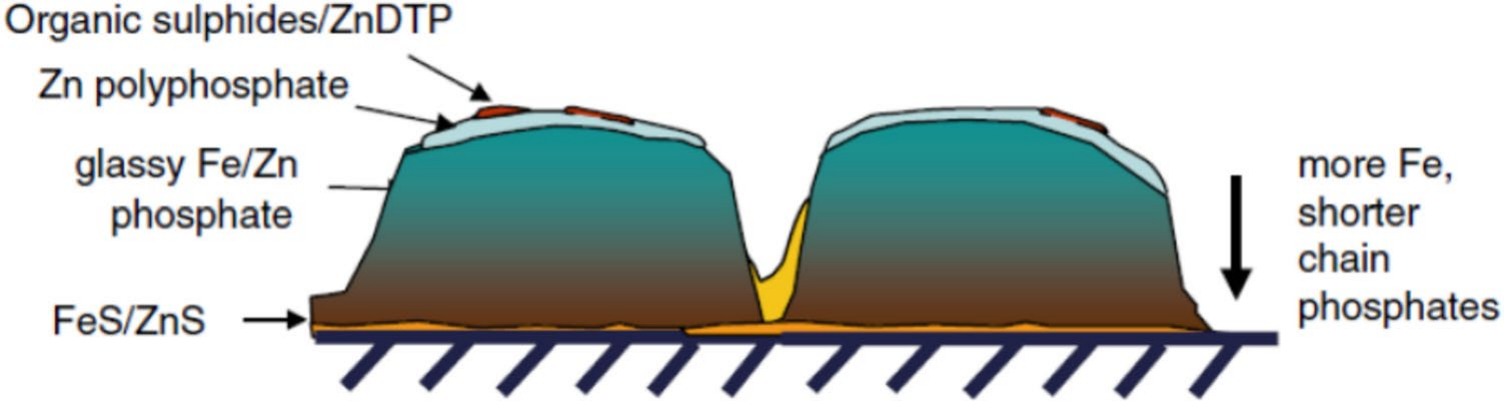
Patchy Structure of ZDDP-derived Tribofilms bonded to a steel surface [2]. Reproduced with permission from the work of Spikes [2], © Springer Nature, 2025

Before the formulation description of the contact problem, it is mentionable that a tribofilm is widely recognized as the thin reacted film on the substrate as a product of tribochemical reactions at the interface driven by the frictional heating and rubbing of contacting surfaces [51]. It can be stress-induced at a temperature that is much lower than that required for thermal films under the effects of rubbing on the reaction kinetics. These thermal effects are out of the scope of the study but shall be investigated in the future considering the essential role of tribochemistry in the performance of tribofilms. Besides, as shown in Fig. 1, ZDDP-derived tribofilms were argued to exhibit a rough, patchy, and graded structure [2]. Each layer was found to exhibit distinct rheological behaviour such that the top layer performs the most fluid-like while the bottom one close to the substrate performs the most solid-like. Notably, the sulphur-rich base layer was reported to perform like a glue binding the substrate with the zinc phosphate layers that constitutes the main bulk of tribofilms. In this work the tribofilm was assumed to behave as a uniform, smooth and homogenous layer to simplify the following formulation as well as simulation.

### Problem Formulation

**Fig. 2:**
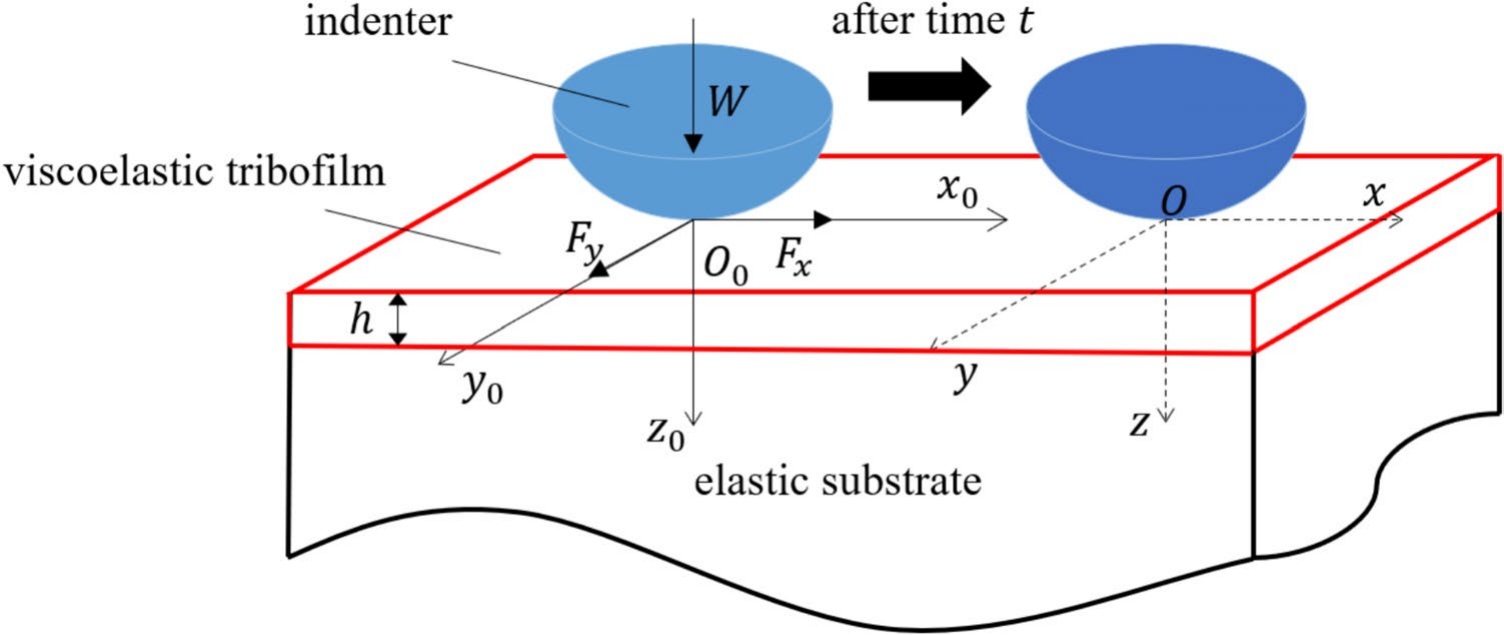
Geometrical description of the contact of a rigid sphere against a viscoelastic layer with uniform thickness $h$ bonded to an elastic substrate under the normal load $W$ and tangential loads ${F}_{x}$ and ${F}_{y}$

The point contact problem of a spherical indenter against a smooth viscoelastic layer bonded to an elastic body is illustrated in Fig. 2. The viscoelastic layer is assumed to be in a uniform thickness $h$ and bonded perfectly to an elastic substrate. Regarding the $z$ coordinate illustrated in Fig. 2, the layer surface and the interface between the layer and substrate are defined as $z=0$ and $z=h$ respectively.

For such layered contact problems, explicit expressions of influence coefficients relating the surface deformations with contact tractions can hardly be derived in a conventional way as those for homogeneous half-space (e.g. via Boussinesq integral [52] and Cerruti’s solutions [53]). However, as suggested by Liu, et al. [54, 55], the problem can be solved by deducing the response functions in the frequency domain first and then using the reverse Fourier transform to obtain the corresponding influence coefficients in the spatial domain. Considering that the correspondence principle was applied to the conversion between elastic and viscoelastic problems, only the derivation of the frequency response functions for elastic layered contact problems is described in detail here to avoid repeated contents.

For an elastic layered contact system, when considering the action of normal pressure $p$ and shear traction in *x* direction ${q}_{x}$, there exist following boundary conditions regarding the stress $\sigma$ at the upper layer surface ($z=0$), the layer is denoted with superscript $1$:

1 $$\sigma_{zz}^{\left( 1 \right)} \left( {x,y,0} \right) = - p\left( {x,y} \right),\,\sigma_{xz}^{\left( 1 \right)} \left( {x,y,0} \right) = q_{x} (x,y),\,\sigma_{yz}^{\left( 1 \right)} \left( {x,y,0} \right) = 0$$

Besides, the following continuous conditions in terms of the stresses and displacements shall be satisfied for the interface between the layer and substrate ($z=h$).

2 $$\begin{gathered} \sigma_{xz}^{\left( 1 \right)} \left( {x,y,h} \right) = \sigma_{xz}^{\left( 2 \right)} \left( {x,y,0} \right),\,\sigma_{yz}^{\left( 1 \right)} \left( {x,y,h} \right) = \sigma_{yz}^{\left( 2 \right)} \left( {x,y,0} \right), \hfill \\ \sigma_{zz}^{\left( 1 \right)} \left( {x,y,h} \right) = \sigma_{zz}^{\left( 2 \right)} \left( {x,y,0} \right), \hfill \\ u_{x}^{\left( 1 \right)} \left( {x,y,h} \right) = u_{x}^{\left( 2 \right)} \left( {x,y,0} \right),\,u_{y}^{\left( 1 \right)} \left( {x,y,h} \right) = u_{y}^{\left( 2 \right)} \left( {x,y,0} \right), \hfill \\ u_{z}^{\left( 1 \right)} \left( {x,y,h} \right) = u_{z}^{\left( 2 \right)} \left( {x,y,0} \right) \hfill \\ \end{gathered}$$

For the substrate denoted with superscript $2$, the stresses and displacements shall vanish at a considerably large distance from the contact surface expressed as:

3 $$\sigma^{\left( 2 \right)} \left( {x,y,\infty } \right) = 0, u^{\left( 2 \right)} \left( {x,y,\infty } \right) = 0$$

The response functions for calculating the displacements derived from contact tractions can be determined in the frequency domain by employing the Papkovich-Neuber potentials (results of the combination of Helmholtz representation and Navier equations) and double Fourier transform. For zero body force, the Papkovich-Neuber potentials $\varphi$ and $\psi ({\psi }_{1},{\psi }_{2},{\psi }_{3})$ are harmonic functions of $x$, $y$, $z$ in the spatial domain. The number of independent harmonic functions can be reduced to three by arbitrarily choosing one of $\psi ({\psi }_{1},{\psi }_{2},{\psi }_{3})$ functions to be zero. As a common practice, here ${\psi }_{2}$ is taken to be zero.

The displacement and stress can be expressed in the following forms as functions of the Papkovich-Neuber potentials:

4 $$\begin{gathered} 2G_{k} u_{i}^{\left( k \right)} = \varphi_{,i}^{\left( k \right)} + x\psi_{1,i}^{\left( k \right)} + z_{k} \psi_{3,i}^{\left( k \right)} - \left( {3 - 4\nu_{k} } \right)\psi_{i}^{\left( k \right)} , \hfill \\ \sigma_{ij}^{\left( k \right)} = \varphi_{,ij}^{\left( k \right)} - 2\nu_{k} \left( {\psi_{1,1 }^{\left( k \right)} + \psi_{3,3}^{\left( k \right)} } \right)\delta_{ij} - \left( {1 - 2\nu_{k} } \right)\left( {\psi_{i,j}^{\left( k \right)} + \psi_{j,i}^{\left( k \right)} } \right) + x\psi_{1,ij}^{\left( k \right)} + z_{k} \psi_{3,ij}^{\left( k \right)} , \hfill \\ \end{gathered}$$

where the index notations $i$ and $j$ have values of $1$, $2$ and $3$ corresponding to $x$, $y$ and $z$ coordinates, respectively, the superscript $k$ represents the layer ($k=1$) or substrate ($k=2$) being referred to, ${G}_{k}$ denotes the shear modulus, ${\nu }_{k}$ denotes the Poisson’s ratio and ${\delta }_{ij}$ denotes the Kronecker delta (${\delta }_{ij}=1$ if $i=j$, or ${\delta }_{ij}=0$ if $i\ne j$).

By applying double Fourier transform, Eq. 4 is transformed into the following form:

5 $$\begin{gathered} \widetilde{{\tilde{u}}}_{i}^{\left( k \right)} = \frac{1}{{2G_{k} }}FT_{xy} \left[ {\varphi_{,i}^{\left( k \right)} + x\psi_{1,i}^{\left( k \right)} + z\psi_{3,i}^{\left( k \right)} - \left( {3 - 4\nu_{k} } \right)\psi_{i}^{\left( k \right)} } \right], \hfill \\ \widetilde{{\tilde{\sigma }}}_{ij}^{\left( k \right)} = FT_{xy} \left[ {\varphi_{,ij}^{\left( k \right)} - 2\nu_{k} \left( {\psi_{1,1 }^{\left( k \right)} + \psi_{3,3}^{\left( k \right)} } \right)\delta_{ij} - \left( {1 - 2\nu_{k} } \right)\left( {\psi_{i,j}^{\left( k \right)} + \psi_{j,i}^{\left( k \right)} } \right) + x\psi_{1,ij}^{\left( k \right)} + z_{k} \psi_{3,ij}^{\left( k \right)} } \right], \hfill \\ \end{gathered}$$

where the hat “$\approx$” and symbol $F{T}_{xy}$ indicate the double Fourier transform operation with respect to $x$ and $y$ directions.

In the frequency domain, the double Fourier-transformed forms of Papkovich-Neuber potentials are expressed as:

6 $$\begin{gathered} \widetilde{{\tilde{\varphi }}}^{\left( k \right)} = A^{\left( k \right)} e^{{ - \alpha z_{k} }} + \overline{A}^{\left( k \right)} e^{{\alpha z_{k} }} ,\,\widetilde{{\tilde{\psi }}}_{1}^{\left( k \right)} = B^{\left( k \right)} e^{{ - \alpha z_{k} }} + \overline{B}^{\left( k \right)} e^{{\alpha z_{k} }} , \hfill \\ \widetilde{{\tilde{\psi }}}_{3}^{\left( k \right)} = C^{\left( k \right)} e^{{ - \alpha z_{k} }} + \overline{C}^{\left( k \right)} e^{{\alpha z_{k} }} \hfill \\ \end{gathered}$$

Considering that the stresses and displacements at a large distance from the substrate surface can be assumed to be zero (expressed as Eq. 3), ${\overline{A} }^{(2)}={\overline{B} }^{(2)}={\overline{C} }^{(2)}=0$. Thus, there are nine unknown parameters in total. The parameter $\alpha$ is determined by $\alpha =\sqrt{{m}^{2}+{n}^{2}}$, where $m$ and $n$ are the transformed frequency variables with respect to $x$ and $y$ in the spatial domain, respectively.

To substitute Eq. 6 into Eq. 5, considering that the stresses and surface displacements are subjected to the nine boundary and interfacial continuity conditions (expressed as Eq. 1, Eq. 2, and Eq. 3), the nine parameters, including ${A}^{(1)}$, ${\overline{A} }^{(1)}$, ${A}^{(2)}$, ${B}^{(1)}$, ${\overline{B} }^{(1)}$, ${B}^{(2)}$, ${C}^{(1)}$, ${\overline{C} }^{(1)}$, ${C}^{(2)}$ can be determined. The closed-form expressions of the influence coefficients in the frequency domain, which are constituted by these parameters, are given in the Appendix A. For the detailed derivation describing the double Fourier transform of all the terms, it can be found in the work of Wang, et al. [56] and Wang and Zhu [57].

It is of note that the computation of the frequency response functions in the frequency domain requires a refined mesh (e.g. a small sampling interval) for reducing the effect of the aliasing phenomenon. For a detailed description of the aliasing and Gibbs phenomena in the Fourier analysis, it can be found in the work of Morrison [58] and Liu and Wang [55]. In this study, a refinement of ${2}^{4}$ times the original mesh was applied to determine the frequency response functions accurately and efficiently. To avoid the singularity problem of the frequency response functions in the origin point ($m=0$, $n=0$), the 64-point Gaussian quadrature integration method was applied to the region around the origin to calculate the response functions.

After determining the frequency response functions for the elastic layered contact problems, those for viscoelastic counterparts can be derived readily by replacing the elastic properties ($\frac{1}{2G}$) with the viscoelastic creep compliance $\phi (t)$. The influence coefficients can then be determined by applying the inverse Fourier transform to the frequency response functions. Once the influence coefficients are obtained, contact tractions and surface displacements in normal and tangential layered contact problems can be determined following the same procedures taken to solve half-space contact problems.

### Algorithm Description

**Fig. 3:**
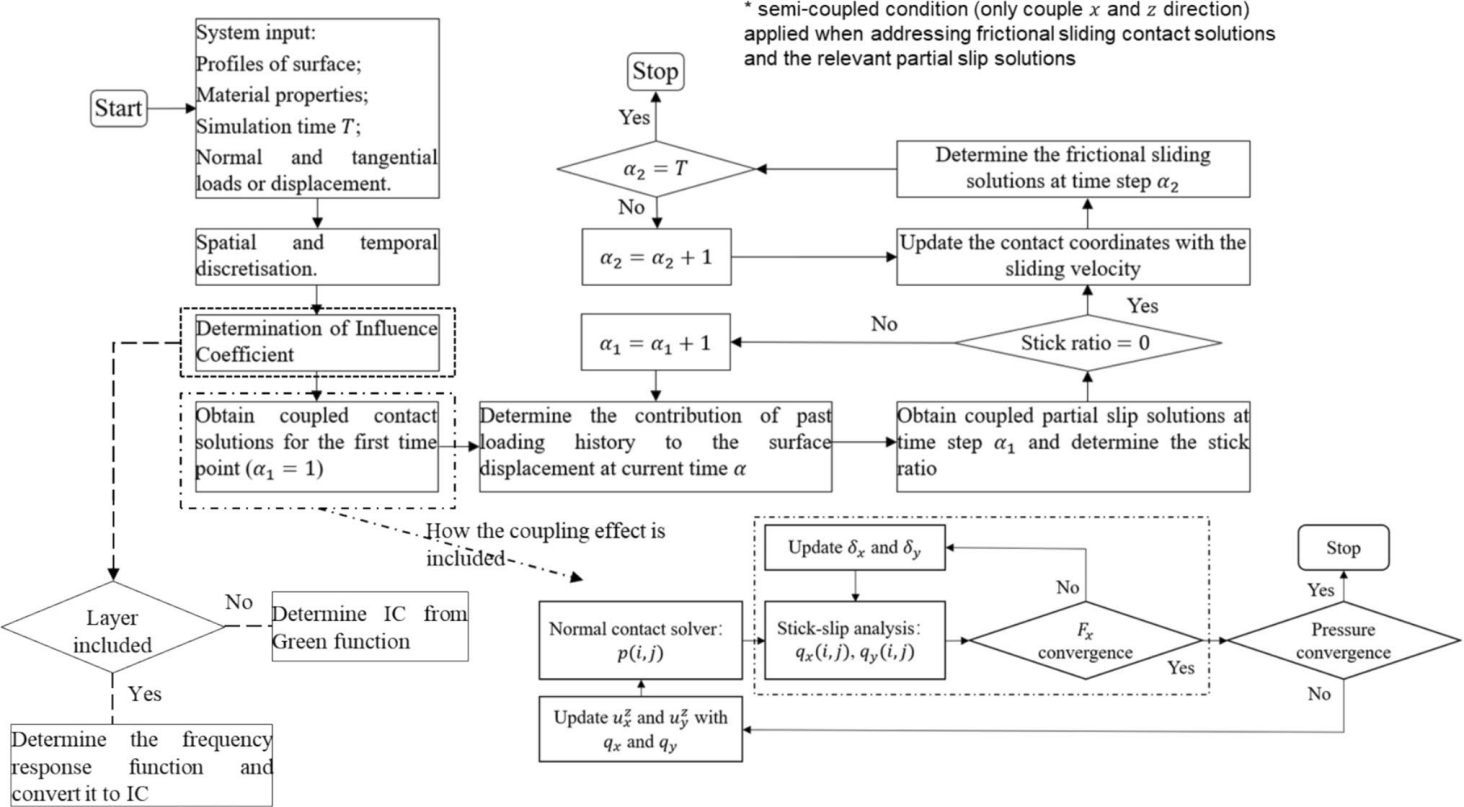
Overview of the algorithm for coupled viscoelastic contact problems (viscoelastic material can either exist as a half-space or layer)

Once the influence coefficients are obtained, contact tractions and surface displacements in normal and tangential layered contact problems can be determined following an algorithm that is similar with that taken to solve half-space contact problems developed previously [59, 60], as illustrated in Fig. 3. Computational techniques, including the discrete convolution fast Fourier transform (DC-FFT) and conjugate gradient method (CGM), were applied to improve the computational efficiency of the algorithm. Hence, instead of describing the details of the algorithms for the layered contact model, here the computational procedures to convert frequency response functions to influence coefficient matrices for layered contact modelling are presented.

To describe the conversion process briefly, it mainly contains the following steps:

1. Determine the frequency response function $F$ in an extended simulation domain;
2. Determine the continuous Fourier-transformed influence coefficient $\widetilde{\widetilde{C}}$ from $F$;
3. Determine the discrete Fourier-transformed influence coefficient $\widehat{\widehat{C}}$ from $\widetilde{\widetilde{C}}$;
4. Apply inverse Fourier transform and wrap-around order to $\widehat{\widehat{C}}$ to determine the influence coefficient $IC$.

To explain each step in detail, assuming that the target computational domain is in the size of ${L}_{1}\times {L}_{2}$ in the spatial dimension, the grid constituted with ${N}_{1}\times {N}_{2}$ elements in the uniform size of ${\Delta }_{x}\times {\Delta }_{y}$ is established to discretize the computational domain. A domain extension factor $\chi$, which is usually the power of $2$ is employed to extend the established mesh system. By employing number of $\chi {N}_{1}\times \chi {N}_{2}$ elements to discretize the extended computational domain, the initial sampling interval in the spatial dimension (${\Delta }_{x}=\frac{{L}_{1}}{{N}_{1}},{\Delta }_{y}=\frac{{L}_{2}}{{N}_{2}}$) is preserved. On the other hand, after adopting an extended computational domain with more mesh elements, the sampling interval in the frequency dimension is reduced, thus alleviating the effects of the aliasing phenomenon since the domain size in the frequency dimension remains as $\frac{2\pi }{{\Delta }_{x}} \times \frac{2\pi }{{\Delta }_{y}}$. The comparison between the computational parameters in the spatial and frequency dimensions is given in Table 1.

**Table 1:** Computational parameters in spatial and frequency dimensions

Parameters	Spatial dimension	Frequency dimension
Length of domain before extension	${L}_{1}\times {L}_{2}$	$\frac{2\pi }{{\Delta }_{x}} \times \frac{2\pi }{{\Delta }_{y}}$
Length of domain after extension	$\chi {L}_{1}\times \chi {L}_{2}$	$\frac{2\pi }{{\Delta }_{x}} \times \frac{2\pi }{{\Delta }_{y}}$
Number of elements before extension	${N}_{1}\times {N}_{2}$	${N}_{1}\times {N}_{2}$
Number of elements after extension	$\chi {N}_{1}\times \chi {N}_{2}$	$\chi {N}_{1}\times \chi {N}_{2}$
Sampling interval before extension	${\Delta }_{x}=\frac{{L}_{1}}{{N}_{1}},{\Delta }_{y}=\frac{{L}_{2}}{{N}_{2}}$	$\frac{2\pi }{{\Delta }_{x}}/{N}_{1}, \frac{2\pi }{{\Delta }_{y}}/{N}_{2}$
Sampling interval after extension	${\Delta }_{x}=\frac{{L}_{1}}{{N}_{1}},{\Delta }_{y}=\frac{{L}_{2}}{{N}_{2}}$	$\frac{2\pi }{{\Delta }_{x}}/({\chi \times N}_{1}), \frac{2\pi }{{\Delta }_{y}}/{(\chi \times N}_{2})$

Once the frequency response function for each surface node at each discretized time step $F(m,n,t)$ in the extended computational domain is determined based on the equations given in Appendix A, instead of applying the inverse Fourier transform, the continuous Fourier-transformed influence coefficients $\widetilde{\widetilde{C}}$ needs to be determined first through the shape function as follows:

7 $$\widetilde{{\widetilde{C}}}\left( {m,n,t} \right) = F\left( {m,n,t} \right) \cdot \widetilde{{\widetilde{Y}}}\left( {m,n} \right),$$

where $Y$ is the shape function and has the following forms in the spatial and frequency domains and $t$ is the index for the discretized time steps ($t=1\dots {N}_{t-1},{N}_{t}$, ${N}_{t}$ is the total number of time points).

8 $$\begin{gathered} Y\left( {x,y} \right) = \left\{ {\begin{array}{ll} {1, \left| x \right| \le \frac{{{\Delta }_{x} }}{2} \,{\text{and}}\, \left| y \right| \le \frac{{{\Delta }_{y} }}{2}} \\ {0,{\text{otherwise}}} \\ \end{array} } \right., \hfill \\ \widetilde{{\widetilde{Y}}}\left( {m,n} \right) = \frac{{4\sin \left( {\frac{{m{\Delta }_{x} }}{2}} \right)\sin \left( {\frac{{n{\Delta }_{y} }}{2}} \right)}}{mn} \hfill \\ \end{gathered}$$

Afterwards, the discrete transformed form of influence coefficient in the Fourier domain $\widehat{\widehat{C}}$ needs to be determined from the continuous Fourier-transformed influence coefficients $\widetilde{\widetilde{C}}$ in the following way:

9 $$\widehat{{\widehat{C}}}\left( {m,n,t} \right) = \frac{1}{{{\Delta }_{x} {\Delta }_{y} }}\mathop \sum \limits_{rx = - AL}^{{r_{x} = AL}} \mathop \sum \limits_{ry = - AL}^{{r_{y} = AL}} \widetilde{{\widetilde{C}}}\left( {\frac{2\pi }{{\chi N_{1} {\Delta }_{x} }}i - \frac{2\pi }{{{\Delta }_{x} }}r_{x} , \frac{2\pi }{{\chi N_{2} {\Delta }_{y} }}i - \frac{2\pi }{{{\Delta }_{y} }}r_{y} ,t} \right),$$

where the hat  represents double discrete Fourier transform operation with respect to $x$ and $y$ directions. $AL$ is the level of aliasing control, and $i$ and $j$ here are the indices for the coordinates of surface nodes in the spatial dimension ($-\frac{{\chi N}_{1}}{2}<i\le \frac{{\chi N}_{1}}{2})$, ($-\frac{{\chi N}_{2}}{2}<j\le \frac{{\chi N}_{2}}{2}$).

After applying the inverse fast Fourier transform ($IFFT$) to the discretized series in the Fourier domain $\widehat{\widehat{C}}$, attention needs to be taken when applying the DC-FFT method in terms of the wrap-around and anti-wrap-around order [55]. The anti-warp-around order is first applied such that the terms corresponding to the negative frequencies are rearranged after the ones corresponding to positive frequencies. After extracting the real parts of the new series of discrete samples ${C}_{temp}$, a matrix $I{C}_{temp}$ in the size of $2{N}_{1}\times 2{N}_{2}$ can be obtained in the following way:

10 $$IC_{temp} \left( {i,j} \right) = Re\left( {C_{temp} \left( {i + \frac{{\chi N_{1} }}{2} - N_{1} ,j + \frac{{\chi N_{2} }}{2} - N_{2} } \right)} \right),$$

where $$i=1\dots 2\times {N}_{1}$$ and $$j=1\dots 2\times {N}_{2}$$.

To apply the wrap-around order to the matrix $I{C}_{temp}$, the conversion from the frequency response function to the influence coefficient is eventually completed. Through converting the discrete linear convolution into discrete cyclic convolution, this transformed influence coefficient matrix $IC$ is ready to be used for the determination of surface deformation in the DC-FFT algorithm [55] with the pressure matrix after the operation of zero-padding.

## Model Validation

Considering that the algorithm developed for the viscoelastic layered contact problems is basically identical to the one that was developed for viscoelastic half-space contact problems, only the layered aspect of the developed model (i.e. the conversion process from frequency response function to influence coefficients) was validated in this section. For the detailed validation in terms of the viscoelastic, coupled partial slip and sliding aspects, readers can refer to the work by Wang, et al. [60] [61].

**Fig. 4:**
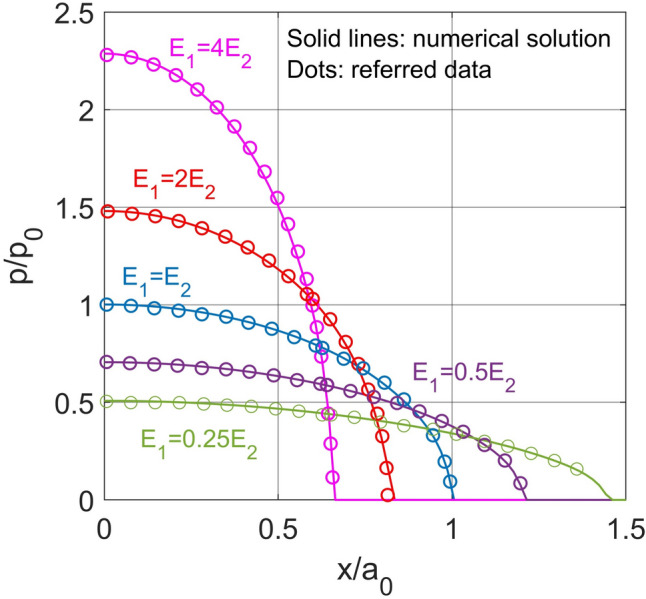
Comparison of nondimensionalized results for elastic layered indentation derived from our model (solid line) and analytical solutions by O’Sullivan and King (scatter). ${E}_{1}$ denotes the elastic modulus of layer, and ${E}_{2}$ denotes the elastic modulus of substrate. [56]

As shown in Fig. 4, the first validation was done by comparing the solutions of the degenerated form of the developed model (i.e. elastic layered contact model) (solid lines) with analytical solutions by O’Sullivan and King [62] (scatters) to elastic layered indentation problems. Here the indentation contact problem between a rigid sphere against an elastic layer bonded to an elastic substrate was simulated. The contact inputs are given in Table 2.

**Table 2:** Parameters used in the validation of elastic layered indentation contact

Parameter	Value	Description (Unit)
$R$	$18$	Radius of sphere ($\text{mm}$)
${E}_{1}$	$\text{52.5,105,210,420,840}$	Elastic modulus of layer ($\text{GPa}$)
${E}_{2}$	210	Elastic modulus of substrate ($\text{GPa}$)
${E}_{3}$	$\infty$	Elastic modulus of indenter ($\text{GPa}$)
${\nu }_{1}/{v}_{2}/{\nu }_{3}$	$0.3$	Poisson’s ratio of layer/substrate/indenter
$W$ ${a}_{0}$ $h/{a}_{0}$ ${p}_{0}$	$20$ $105.373$ $1$ $860$	Input normal load ($\text{N}$) Hertzian contacting radius (μm) Nondimensionalized layer thickness Hertzian peak normal pressure ($\text{MPa}$)

The computational domain is set to be $2{a}_{0}\times 2{a}_{0}$ to accommodate the variation of contacting area under the effects of layers with different properties. The domain is discretised with $256\times 256$ nodes. By varying the ratio of the layer modulus ${E}_{1}$ to substrate modulus ${E}_{2}$ as presented in Table 2, different pressure distributions are observed for elastic layered indentation problems. As shown in Fig. 4, the peak pressure $p$ increases with the modulus ratio while the contacting radius ${a}_{0}$ responds oppositely. Good agreement can be found between the solution derived from our model (solid lines) and analytical solutions (dots) proposed by O’Sullivan and King [62].

**Fig. 5:**
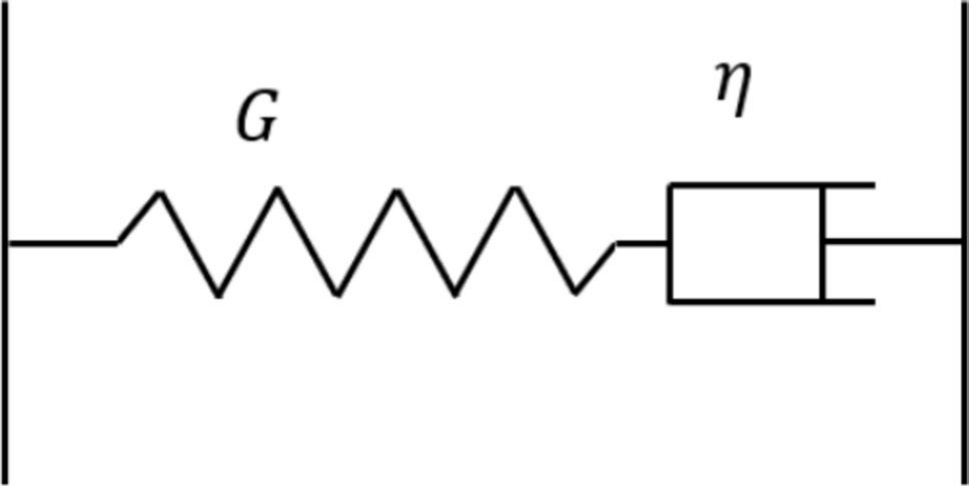
Structure of a Maxwell model: a linear elastic spring with modulus $G$ in series with a dashpot containing a Newtonian fluid (viscosity $\eta$)

To validate the successful application of the elastic–viscoelastic correspondence principle to viscoelastic layered problems, another test was conducted. This test simulated the indentation of a rigid sphere against a viscoelastic layer bonded to an elastic substrate. Two extreme cases were considered by specifying significantly large and small dimensionless layer thicknesses ($h/{a}_{0}$), respectively. Here, the parameter ${a}_{0}$ represents the Hertzian contacting radius for the indentation problem of the rigid sphere against the elastic substrate without the viscoelastic layer. The viscoelastic material was modelled using a Maxwell model, which is constituted by a spring in series with a dashpot as illustrated in Fig. 5. A Maxwell model mathematically expresses the mechanical properties of materials, including relaxation modulus $\Psi (t)$ and creep compliance $\Phi (t)$, as follows:

11 $$\Psi \left( t \right) = G \cdot \exp \left( { - \frac{t}{\tau }} \right),$$

12 $$\Phi \left( t \right) = \frac{1}{G} + \frac{t}{\eta },$$

where $\tau$ is known as the relaxation time of materials and is determined as the ratio of the dashpot viscosity $\eta$ to the spring modulus $G$. The parameters for the established Maxwell model and other relevant contact inputs are given in Table 3.

**Table 3:** Parameters used in the validation of viscoelastic layered indentation contact

Parameter	Value	Description (unit)
$R$	3.5	Radius of sphere ($\text{mm}$)
$G$	$80.77$	Shear modulus of the spring in Maxwell model ($\text{GPa})$
$\eta$	$80.77$	Viscosity of the dashpot in Maxwell model ($\text{GPa s}$)
${E}_{2}$	210	Elastic modulus of substrate ($\text{GPa}$)
${E}_{3}$	$\infty$	Elastic modulus of indenter ($\text{GPa}$)
${\nu }_{1}/{\nu }_{2}/{\nu }_{3}$	$0.3$	Poisson’s ratio of the layer/substrate/indenter
$W$	$100$	Input normal load ($\text{N}$)
${a}_{0}$	$104.388$	Hertzian contacting radius (μm)
$h/{a}_{0}$	$1\times {10}^{4}, 1\times {10}^{-5}$	Nondimensionalized thickness of viscoelastic layer
${a}_{v0}$	$131.5206$	Initial contacting radius for viscoelastic half-space contact (μm)
${p}_{0}$	$4.3817$	Hertzian peak pressure ($\text{GPa}$)
${p}_{v0}$	$2.7603$	Initial peak pressure for viscoelastic half-space contact ($\text{GPa}$)

**Fig. 6:**
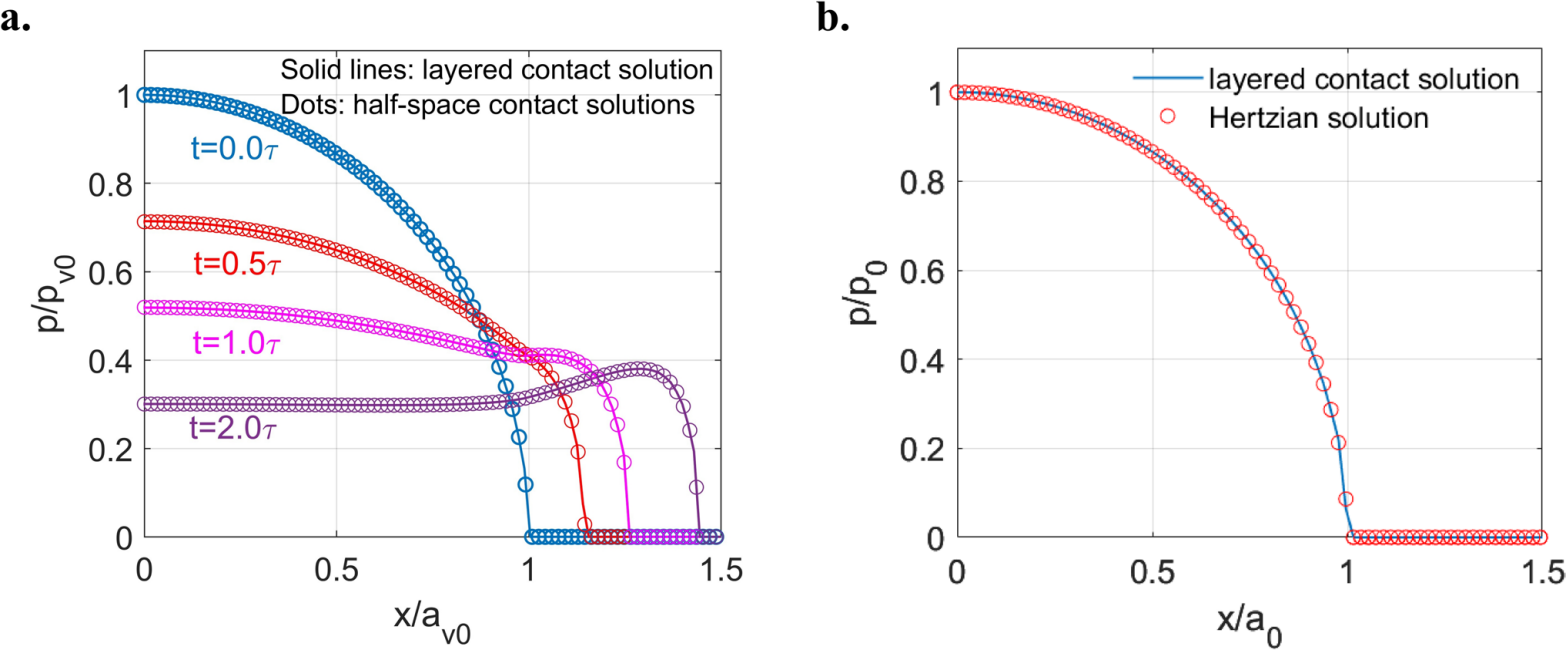
Comparison between the nondimensionalized solutions of viscoelastic layered indentation problem with different layer thicknesses (solid line) and half-space contact problems (scatter): **a** $\frac{h}{{a}_{0}}=1\times {10}^{4}$ and **b** $\frac{h}{{a}_{0}}=1\times 1{0}^{-5}$

For this case, the computational domain is set to be $2{a}_{v0}\times 2{a}_{v0}$ to accommodate the creep of viscoelastic materials under normal loads. This domain is discretised by $256\times 256$ nodes. Besides, the simulation time is set to be $2\tau$, which is discretised by 41 time steps. As shown in Fig. 6a, the contact solutions of the half-space contact (viscoelastic material as the half-space) and layered contact fit together when the viscoelastic layer is extremely thick ($\frac{h}{{a}_{0}}=1\times {10}^{4}$). To highlight the time dependency of materials, the parameters used to nondimensionalize the solutions shown in Fig. 6 (a), including ${a}_{v0}$ and ${p}_{v0}$, are the contacting radius and the peak normal pressure respectively when the viscoelastic contact initialises $(t=0)$. On the other hand, there tends to be no time dependency for the contact solutions when the viscoelastic layer is considerably thin ($\frac{h}{{a}_{0}}=1\times {10}^{-5}$) as illustrated in Fig. 6 (b). Besides, the solution is extremely close to the Hertzian half-space solution (elastic material as the half-space). The layered contact solutions are nondimensionalized by the Hertzian solutions (${a}_{0}$ and ${p}_{0}$) to highlight the effects of the layer. Unless otherwise specified, the half-space and layered contact solutions presented hereinafter are nondimensionalized in the same way, respectively.

The good agreement between the simulation results derived from our model and analytical solutions validates as best possible the viscoelastic layered aspect of the developed model.

## Contact Analysis on the Effects of a Viscoelastic ZDDP-Derived Tribofilm Layer

**Fig. 7:**
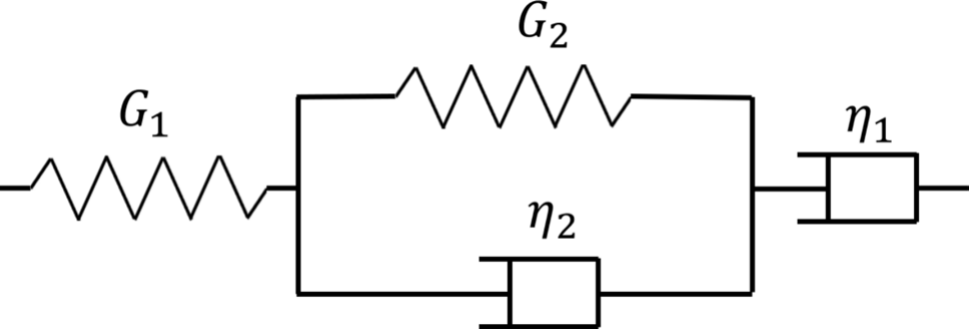
Structure of the Burgers material model used to characterise the creep compliance of the ZDDP-derived tribofilm: $G$ is the modulus of the linear spring and $\eta$ is the viscosity of the dashpot

The validated model can now be extended to study the response of a ZDDP-derived tribofilm layer. To characterize the linear viscoelastic behaviour of the ZDDP-derived tribofilm, as proposed by Dorgham, et al. [25], a Burgers material model, which has a structure illustrated in Fig. 7 and can be mathematically expressed by the four-term Eq. 13, was employed.

To describe the experimental work by Dorgham, et al. [25] briefly, instead of studying tribofilm properties at the end of a rubbing test after the tribofilm had fully formed, they utilized an in-situ atomic force microscopy (AFM) set-up in a high-temperature liquid cell to generate ZDDP-derived tribofilm. The films were generated by rubbing the AFM tip (Diameter $D\approx 150 \ \text{nm}$) against a steel substrate in a liquid medium of poly-$\alpha$-olefin (PAO) base oil containing ZDDP additive under controlled high-temperature and high-pressure conditions. The AFM operated in a standard contact mode using a multi-pass and bidirectional raster scanning with a predetermined number of lines. The scanning lines were found to play a crucial role in the tribofilm morphology such that a high density of scanning lines leads to a congested and continuous morphology while a low density yields a nanostructured tribofilm distinguished by distinctive line features. The nanoscale viscosity of the formed tribofilms was then quantified using a creep method, where a constant stress was applied via the AFM tip. The creep compliance was calculated based on the ratio of the strain (normalized change in tribofilm area) to stress (shear stress).

According to Dorgham, et al. [25], it can be observed that after a relatively long time, the viscosity of tribofilm tends to play a dominant role in its creep behaviour as the creep compliance keeps increasing with time following a linear trend. This implies that the ZDDP-derived tribofilm is extremely fluid-like such that its creep compliance can hardly reach a steady state. According to the findings reported in our previous studies regarding the effects of the rheological behaviour of viscoelastic materials [60], pressure spikes on contacting edges shall be expected in the following tests, especially when the tribofilm exists as a half-space.

13 $$\Phi \left( t \right) = \frac{1}{{G_{1} }} + \frac{1}{{G_{2} }}\left( {1 - \exp \left( { - \frac{{G_{2} }}{{\eta_{2} }}t} \right)} \right) + \frac{1}{{\eta_{1} }}t$$

**Fig. 8:**
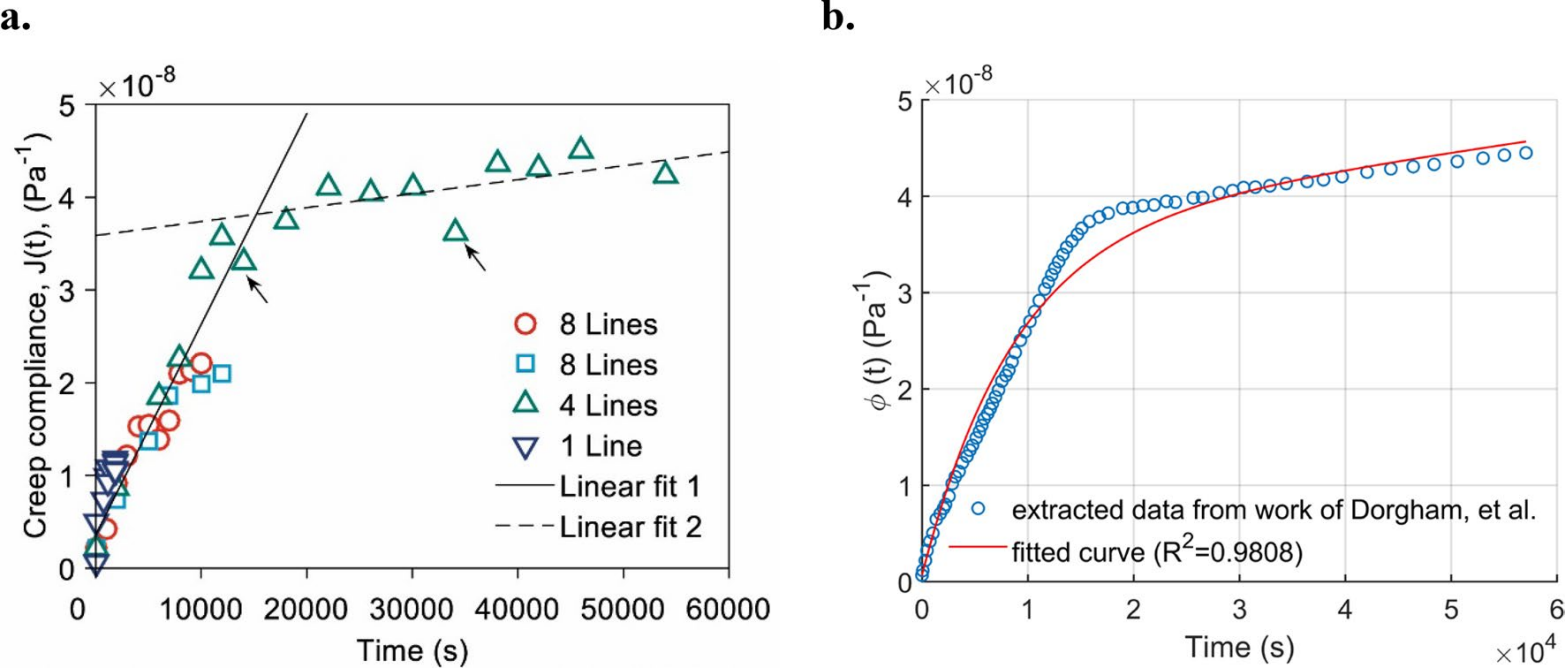
**a** Creep compliance of the ZDDP-derived tribofilm reported by Dorgham, et al. [25], the term “Lines” in the legend stands for the scanning lines in the AFM experiments. Reproduced with permission from Ref. [25], © Elsevier B. V., 2025. and **b** Four-term trend line generated through curve fitting (${R}^{2}=0.9808$)

By applying curve fitting on several selected data points extracted from Fig. 8 (a) of the work of Dorgham, et al. [25], and incorporating additional data points proximal to the linear-fit trend lines originally generated by Dorgham to refine the curve fitting effects, a trend line, which is mathematically expressed as Eq. 14, is obtained as depicted in Fig. 8 (b). A close correlation can be found between the trend line and the referred data (${R}^{2}=0.9808$) as observed in Fig. 8 (b). Notably, this fitting work was conducted based on the built-in curve fitting toolbox in MATLAB, where Eq. 13 is used as the input custom equation.

14 $$\phi \left( t \right) = \frac{1}{1.5} + \frac{1}{0.02778}\left[ {1 - \exp \left( { - \frac{0.02778}{{241.8}} \times t} \right)} \right] + \frac{1}{6297.2}t\,\left( {\frac{1}{{{\text{GPa}}}}} \right)$$

To study the effects of ZDDP-derived tribofilms with the aforementioned viscoelastic property, it is crucial to specify the typical contact conditions encountered in realistic applications. While the developed model can handle rough frictional contact problems (e.g. partial slip or sliding contact), our previous study [59] demonstrated that for steady sliding contact, the role of dry contact friction can be neglected due to its minimal impact on the steady solutions. Additionally, including dry contact friction significantly increases the computational cost due to the coupling between shear tractions and pressures during partial slip analysis. For instance, on a desktop computer with 6 cores, solving an uncoupled partial slip contact problem of elastic rough surfaces with a resolution of 512 $\times$ 512 nodes takes approximately 140 s [63], while incorporating coupling effects increases the computational time to 300 s [61]. This extra computational cost will be significantly higher for viscoelastic contact problems.

In real-world applications, such as gears and bearings, tribofilms perform under sustained sliding conditions. These films form dynamically at contact interfaces, with their formation and removal rates balancing over time to eventually maintain a steady layer thickness. This mechanism ensures a continuous protective surface layer that mitigates direct contact between bodies in the long run [2, 27, 64]. By assuming frictionless contact, the balance between the computational efficiency and ability to capture the critical viscoelastic effects of the tribofilm under steady sliding conditions is achieved. To closely resemble the realistic scenarios, the applied load, geometry and materials of the two contacting bodies were selected based on the tribotest conducted by Ghanbarzadeh, et al. [65].

Regarding other properties of the tribofilm, considering that it is usually very thin as it works in the boundary lubrication regime, the film thickness should be in the order of nanometers. Here the tribofilm thickness is assumed to be time-independent *h* = 150 nm, which is a typical value according to the experimental study of Ghanbarzadeh, et al. [65] and Dorgham, et al. [25]. However, as mentioned before, the formation and removal of the tribofilm occur simultaneously in practice, the thickness of the tribofilm should vary with time before its generation process reaches a relatively steady state. The variation of the layer thickness as well as its material property may have synergistic effects on the contact solutions. To our best knowledge, the transient contact of a tribofilm with a time-dependent layer thickness cannot yet be simulated with the developed viscoelastic layered model alone as the time-dependent influence coefficient cannot describe the surface displacement induced by the unit pressure while the material property and layer thickness vary simultaneously. Thus, the following studies focus on the effects of the tribofilm when its thickness has already reached a steady state while the transient effects of tribofilm formation and removal are left for future work.

The tribofilm is assumed to have a constant Poisson’s ratio $(\nu =0.3)$, which is within a reasonable range according to the study of Matori, et al. [66]. Given that the tribofilm itself has no steady state in terms of its creep compliance as mentioned above, the simulated contact time starts with 120 min in the following simulations for the sake of computational efficiency. Such a test duration is commonly adopted in experimental work about ZDDP-derived tribofilms (e.g. the study of Ghanbarzadeh, et al. [65, 67]). All these contact parameters are given in Table 4. To note, the simulated contact time was adjusted according to the time when the solution becomes steady for the problems under different operating conditions.

**Table 4:** Parameters used in the simulation of ZDDP-derived tribofilm

Parameters	Value	Description (Unit)
$D$	19.05	Diameter of sphere ($\text{mm}$)
${E}_{2}$	210	Elastic modulus of substrate ($\text{GPa}$)
${E}_{3}$	$210$	Elastic modulus of indenter ($\text{GPa}$)
${\nu }_{1}/{\nu }_{2}/{\nu }_{3}$	$0.3$	Poisson’s ratio of the layer/substrate/indenter
$W$	$60$	Input normal load ($\text{N}$)
${a}_{0}$	$155$	Hertzian contacting radius (μm)
${p}_{0}$	$1.19$	Hertzian peak pressure (G$\text{Pa}$)
$T$	120	Simulation time ($\text{min}$)
$h$	150	Thickness of tribofilm $(\text{nm})$

### Indentation Contact

**Fig. 9:**
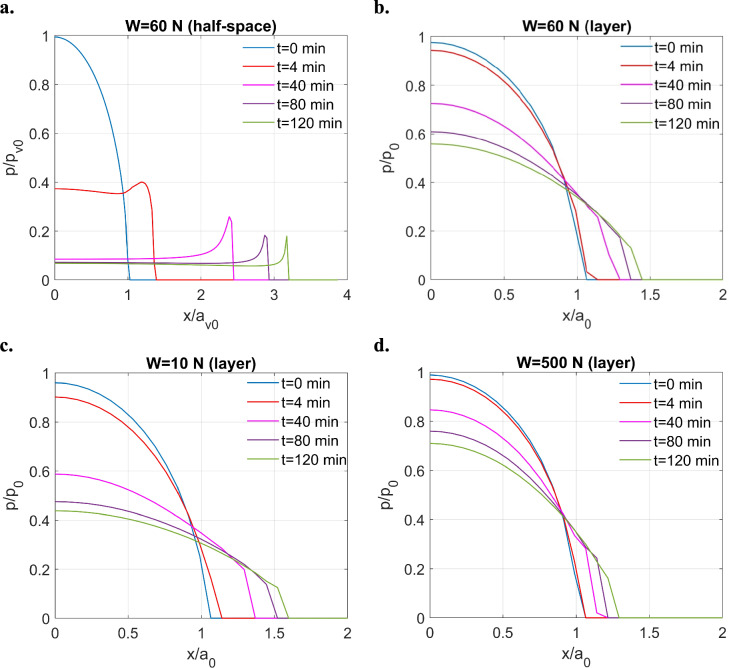
Pressure distributions for ZDDP tribofilm indentation problem under fixed normal loads: **a** half-space ($W= 60 \ \text{N}$), **b** layered ($W= 60 \ \text{N}$), **c** layered ($W= 10 \ \text{N}$), and **d** layered ($W= 500 \ \text{N}$)

Before the sliding simulation, the role played by the tribofilm under indentation conditions was first investigated to evaluate its time-dependent viscoelastic behaviour under quasi-static conditions. As a reference, the first test was conducted by assuming that the tribofilm has an infinite thickness such that it behaves as a half-space. This half-space case highlights the intrinsic viscoelastic behaviour of the tribofilm without the interference from layer thickness effects or substrate properties, which helps the following analysis of simulation outcomes. The simulation domain for this case is set to be $1.1{a}_{max}\times 1.1{a}_{max}$ to accommodate the creep of viscoelastic materials under normal loads, where ${a}_{max}$ is the contacting radius when the viscoelastic contact reaches the end of simulation window *t* = 120 min. This computational domain is discretised with $256\times 256$ nodes. For the contact between a steel sphere-shape indenter against the flat viscoelastic half-space under a normal load, the variation of the pressure distribution with time is shown in Fig. 9a. As expected, when existing as a half-space body, the tribofilm behaves as an extremely fluid-like material. This is indicated by the phenomenon that the load keeps being distributed to both edges of the increasing contacting area, leading to sharp pressure spikes [60].

To switch to the layered indentation problem (the contact between a steel sphere indenter against the tribofilm bonded to a flat steel substrate), the computational domain is decreased to $$0.8{a}_{max}\times 0.8{a}_{max}$$ as the creep phenomenon is expected to be less significant for the layered case. Under the same normal load, as illustrated in Fig. 9b, the compliant viscoelastic layer results in the decrease of the contact pressure and increase of contacting area. However, the pressure spikes cannot be observed in this case, which suggests that the combination of the tribofilm and carbon steel substrate now performs more solid-like compared with the tribofilm half-space. Such rheological behaviour can be modified by varying the input load when the layer thickness remains with time. As illustrated in Fig. 9c and d, more fluid-like contact behaviour (e.g. significantly reduced pressure profiles) is achieved for the current viscoelastic layered contact problem when the normal load decreases from 500 to 10 N. This contact behaviour is related to the ratio of the layer thickness to the Hertzian contacting radius ($$h/{a}_{0})$$ given in Table 5. The contact response of the combined substance shall keep approaching that of a viscoelastic half-space when the dimensionless layer thickness increases.

**Table 5:** Variation of the dimensionless layer thickness $h/{a}_{0}$ with the constant contact input in layered indentation problem

Input in layered indentation problem	$h/{a}_{0}$	${p}_{0}$ (GPa)
*W* = 10 N	$0.0018$	0.657
*W* = 60 N	$9.6853\times {10}^{-4}$	1.2
*W* = 500 N	$4.7772\times {10}^{-4}$	2.42
${\delta }_{z}$ = 0.7796 μm	$0.0096$	0.665
${\delta }_{z}$ = 2.5468 μm	$9.6308\times {10}^{-4}$	1.2
${\delta }_{z}$ = 10.4002 μm	$4.7658\times {10}^{-4}$	2.43

Apart from the creep phenomenon under a constant normal load, the stress relaxation phenomenon under a constant displacement for the ZDDP-derived tribofilm is investigated here. The rigid body displacements $({\delta }_{z})$ of the first time point determined in the former four creep tests are used as the input for the following relaxation tests, correspondingly.

**Fig. 10:**
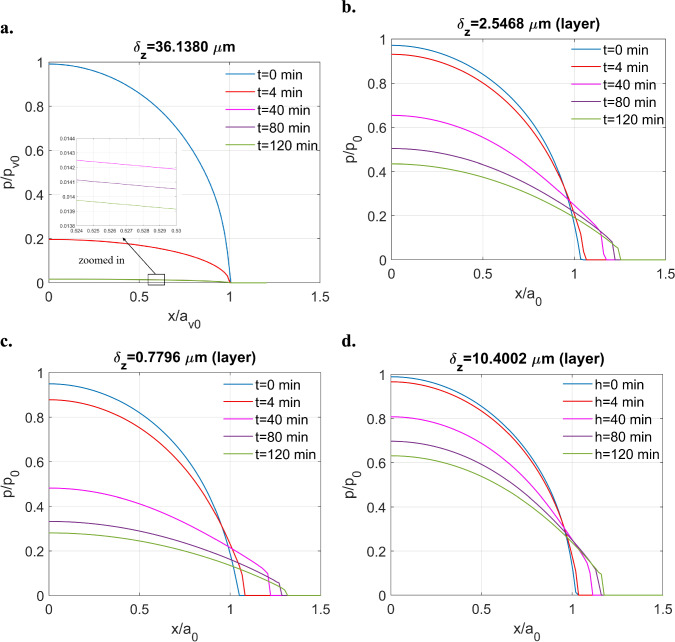
Solutions to the indentation problem of the ZDDP tribofilm in different forms under a fixed normal displacement: **a** half-space contact $\left( {\delta_{z} = 36.1380\, \upmu {\text{m}}} \right)$, **b** layered contact $\left( {\delta_{z} = 2.5468\, \upmu {\text{m}}} \right)$, **c** layered contact $\left( {\delta_{z} = 0.7796\, \upmu {\text{m}}} \right),$ and **d** layered contact $(\delta_{z} = 10.4002 \,\upmu {\text{m}})$

The half-space contact of the ZDDP-derived tribofilm under a fixed normal displacement $(\delta_{z} = 36.1380 \,\upmu {\text{m}})$ is first simulated as a reference. The simulation domain for this case is set is to be $1.2{a}_{v0}\times 1.2{a}_{v0}$, which is discretised by $256\times 256$ nodes. As shown in Fig. 10a, a typical stress relaxation phenomenon, including the constant contacting area and significant decrease of pressure with time, is observed when the tribofilm performs as a half-space.

Notable results are observed as illustrated in Fig. 10b when the tribofilm is modelled as a finite-thickness layer under a specific displacement $(\delta_{z} = 2.5469\, \upmu {\text{m}})$. For the layered case, the simulation domain is set to be $2{a}_{0}\times 2{a}_{0}$ discretised by $256\times 256$ nodes. The contacting area increases over time although it tends to be steady eventually. By increasing the surface displacement, thereby reducing the dimensionless layer thickness as given in Table 5, the stress relaxation effect becomes less pronounced as depicted in Fig. 10c and d, where the displacement increases from 0.7796 to 10.4002 μm, respectively. In all layered cases, an increase in the contacting areas was observed. These findings suggest that the combination of the tribofilm and substrate can demonstrate unique contact behaviour under a constant displacement, differing from the typical responses of purely viscoelastic or elastic solids.

**Fig. 11:**
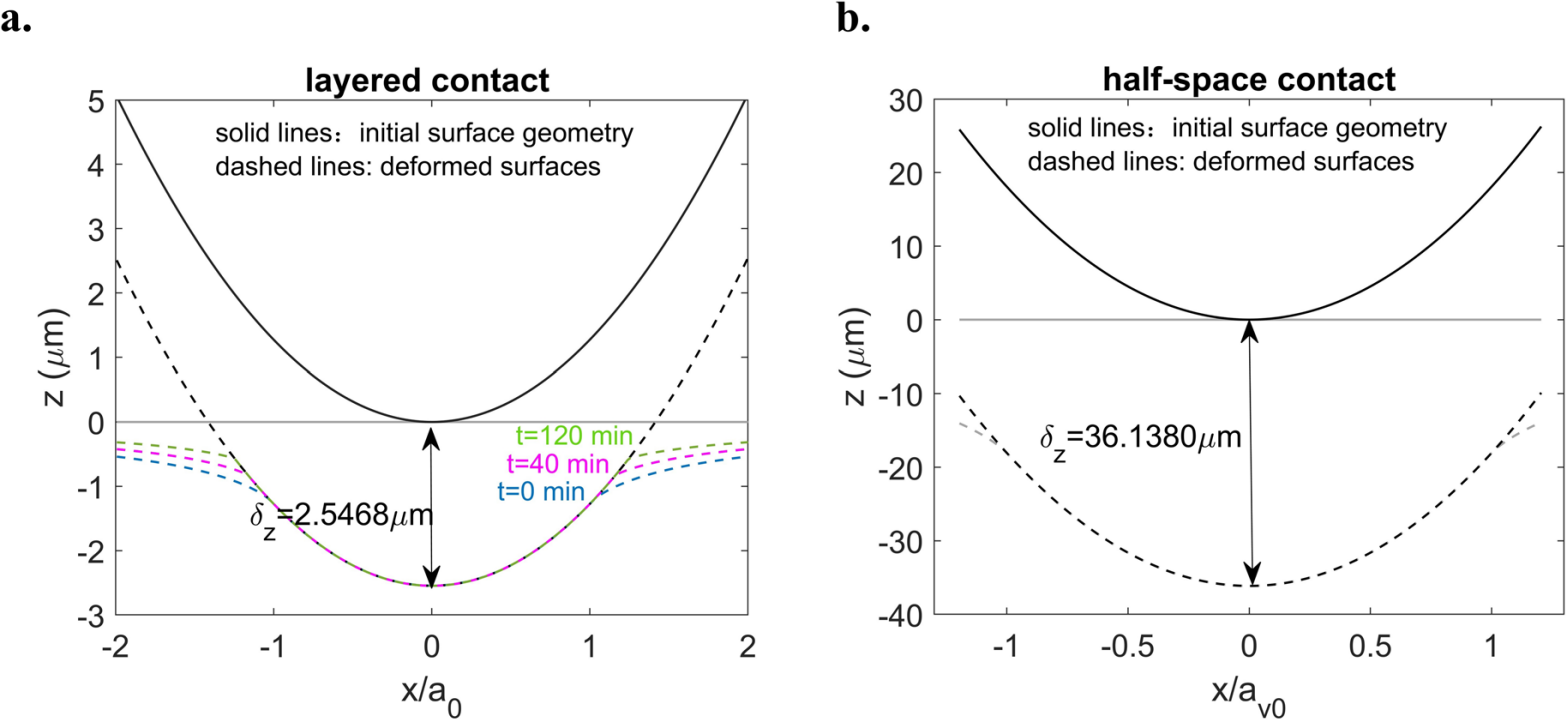
Surface deformation of the contacting bodies under different conditions **a** layered contact $\left( {\delta_{z} = 2.5468 \,\upmu {\text{m}}} \right),$ and **b** half-space contact $\left( {\delta_{z} = 36.1380 \,\upmu {\text{m}}} \right)$

To investigate why the contact area increases over time under a constant surface displacement in the layered tribofilm contact, the surface deformations for the half-space case and layered case are plotted in Fig. 11. These cases are simplified to the indentation of a rigid sphere against a flat half-space with equivalent material properties derived from all the contacting bodies. For the layered case, the contact becomes increasingly conformal over time, even though the surface displacement remains constant as illustrated in Fig. 11a. In contrast, the contact geometry for the half-space case remains unchanged over time, as illustrated in Fig. 11b. This difference can be attributed to the confinement by the substrate. It limits vertical deformation of the viscoelastic tribofilm, causing the tribofilm to flow laterally over time. This lateral flow leads to an increase in contacting area.

**Fig. 12:**
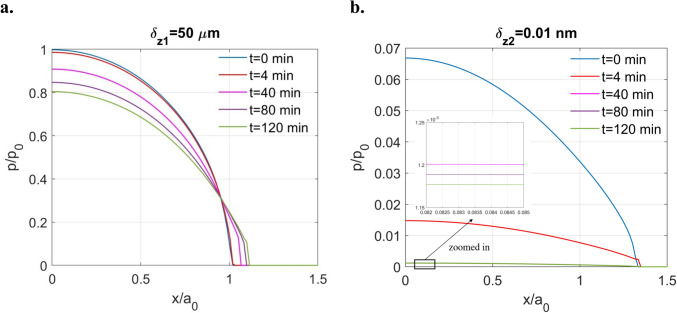
Pressure distribution under extreme displacement: **a** $\delta_{z1} = 50 \,\upmu {\text{m}}$ and **b** ${\delta }_{z2}=0.01 \ \text{nm}$

To validate this idea, two extreme cases under constant surface displacement ($\delta_{z1} = 50 \ \upmu {\text{m}}$ and ${\delta }_{z2}=0.01 \ \text{nm}$) were tested. As shown in Fig. 12a, when the displacement is extremely large $(\delta_{z1} = 50 \,\upmu {\text{m}})$, the tribofilm initially has minimal effect on contact solutions, with the initial pressure closely matching the Hertzian solution. However, the contacting area starts to exhibit time dependency as time progresses, indicating the lateral flow of tribofilm. On the other hand, when the surface displacement is extremely small (${\delta }_{z2}=0.01 \ \text{nm}$) even compared with the ultra-thin tribofilm, the contacting area tends to remain constant while the pressure relaxes and reaches the steady state within a short time. This behaviour resembles a half-space contact, as the tribofilm tends to behave as if unbounded by the substrate under such a low displacement, which is in consistency with our prior understanding.

It is important to note that this phenomenon is influenced by several factors, including the rheological properties of the material, layer thickness, specified surface displacement, and substrate stiffness. When the layered material behaves more solid like (high resistance to flow), the layer thickness is much greater than the specified surface displacement (insignificant confinement effect), and the substrate exhibits mechanical properties similar to the layer (contact response similar to a bulk material), the increase in the contacting area becomes negligible. For instance, Wallace et al. [49] conducted a test simulating the indentation of a rigid sphere against a thick polymethyl methacrylate (PMMA) coating bonded to the carbon steel substate under a comparatively low surface displacement, where no increase in contacting area was observed.

### Sliding Contact

Based on the previously developed algorithm for the sliding contact of viscoelastic half-space bodies [59], the sliding motion is achieved by keeping the indenter (sphere) static while moving the counter body (tribofilm and substrate) in the direction opposite to the sliding motion. The sliding speed correlates with the pixel width and time interval of the established system such that for each time step, the counter body is moved in the distance of certain amount of pixel width. This leads to one limitation of the developed model such that when a high sliding speed is specified, an extremely large simulation domain needs to be generated, within which the actual contacting area may take up a small amount and this can lead to serious discretization error. On the other hand, an extremely small simulation domain needs to be specified for a considerably low-speed sliding contact, which struggles to accommodate the real contacting area. The issue of the discretization of the spatial domain can be avoided by adjusting the way the temporal domain is discretized. However, this action may undermine computational efficiency from another perspective when a great number of time steps are used. A compromise needs to be made to ensure a balance between the computational accuracy and efficiency.

To facilitate the following sliding simulation and result comparison, where varying speeds are specified for different cases, the computational domains are set relative to the same specific parameter ${a}_{0}^{*}$, which is the contact radius of the tribofilm layered contact $(h=150 \ \text{nm})$ under a low load $W=1 \ \text{N}$ when the viscoelastic contact initializes. The detailed computational parameters for each case, including the size of computational domain (denoted as ${L}_{1}\times {L}_{2}$), pixel width in $x$ direction (denoted as ${\Delta }_{x}$), total simulation time (denoted as $T$) and time interval (denoted as ${\Delta }_{t}$) are shown in Table 6. Notably, as the pixel width in $y$ direction is identical to that in $x$ direction, only that in $x$ direction is described here.

**Table 6:** Computational parameters employed for simulating viscoelastic sliding contact

Contact conditions	${L}_{1}\times {L}_{2}$	${\Delta }_{x}$ ($\upmu \text{m}$)	$T$ ($\text{min}$)	${\Delta }_{t}$ ($\text{s}$)
$W= 60 \ \text{N}$ $v= 0.4028 \ \upmu {\text{m}}/{\text{s}}$ half-space contact	$16.5{a}_{0}^{*}\times 16.5{a}_{0}^{*}$	19.3324	240	48
${\delta }_{z}=36.1380 \ \upmu {\text{m}},$ $v=0.4028 \ \upmu {\text{m}}/{\text{s}}$ half-space contact	$16.5{a}_{0}^{*}\times 16.5{a}_{0}^{*}$	19.3324	240	48
$W=1 \ \text{N},$ $v=0.0336 \ \upmu {\text{m}}/{\text{s}}$ layered contact	$1.375{a}_{0}^{*}\times 1.375{a}_{0}^{*}$	1.6110	240	48
$W=60 \ \text{N},$ $v=0.0336 \ \upmu {\text{m}}/{\text{s}}$ layered contact	$1.375{a}_{0}^{*}\times 1.375{a}_{0}^{*}$	1.6110	240	48
$W=1 \ \text{N},$ $v=0.4028 \ \upmu \text{m}/\text{s}$ layered contact	$16.5{a}_{0}^{*}\times 16.5{a}_{0}^{*}$	19.3324	240	48
$W=60 \ \text{N},$ $v=0.4028 \ \upmu \text{m}/\text{s}$ layered contact	$16.5{a}_{0}^{*}\times 16.5{a}_{0}^{*}$	19.3324	240	48
$W=1 \ \text{N},$ $v=0.8055 \ \upmu \text{m}/\text{s}$ layered contact	$1.375{a}_{0}^{*}\times 1.375{a}_{0}^{*}$	1.6110	2	2
$W=60 \ \text{N},$ $v=0.8055 \ \upmu \text{m}/\text{s}$ layered contact	$1.375{a}_{0}^{*}\times 1.375{a}_{0}^{*}$	1.6110	10	2
$W=1 \ \text{N},$ $v=6.441 \ \upmu \text{m}/\text{s}$ layered contact	$1.375{a}_{0}^{*}\times 1.375{a}_{0}^{*}$	1.6110	1	0.25
$W=60 \ \text{N},$ $v=6.441 \ \upmu {\text{m}}/{\text{s}}$ layered contact	$1.375{a}_{0}^{*}\times 1.375{a}_{0}^{*}$	1.6110	2	0.25
$W=1 \ \text{N},$ $v=12.89 \ \upmu {\text{m}}/{\text{s}}$ layered contact	$1.375{a}_{0}^{*}\times 1.375{a}_{0}^{*}$	1.6110	1	0.1250
$W=60 \ \text{N},$ $v=12.89 \ \upmu {\text{m}}/{\text{s}}$ layered contact	$1.375{a}_{0}^{*}\times 1.375{a}_{0}^{*}$	1.6110	1	0.1250
$W=1 \ \text{N},$ $v=1.661 \ {\text{mm}}/{\text{s}}$ layered contact	$6.875{a}_{0}^{*}\times 6.875{a}_{0}^{*}$	8.0551	1/6	0.005
$W=60 \ \text{N},$ $v=1.661 \ {\text{mm}}/{\text{s}}$ layered contact	$6.875{a}_{0}^{*}\times 6.875{a}_{0}^{*}$	8.0551	1/6	0.005

**Fig. 13:**
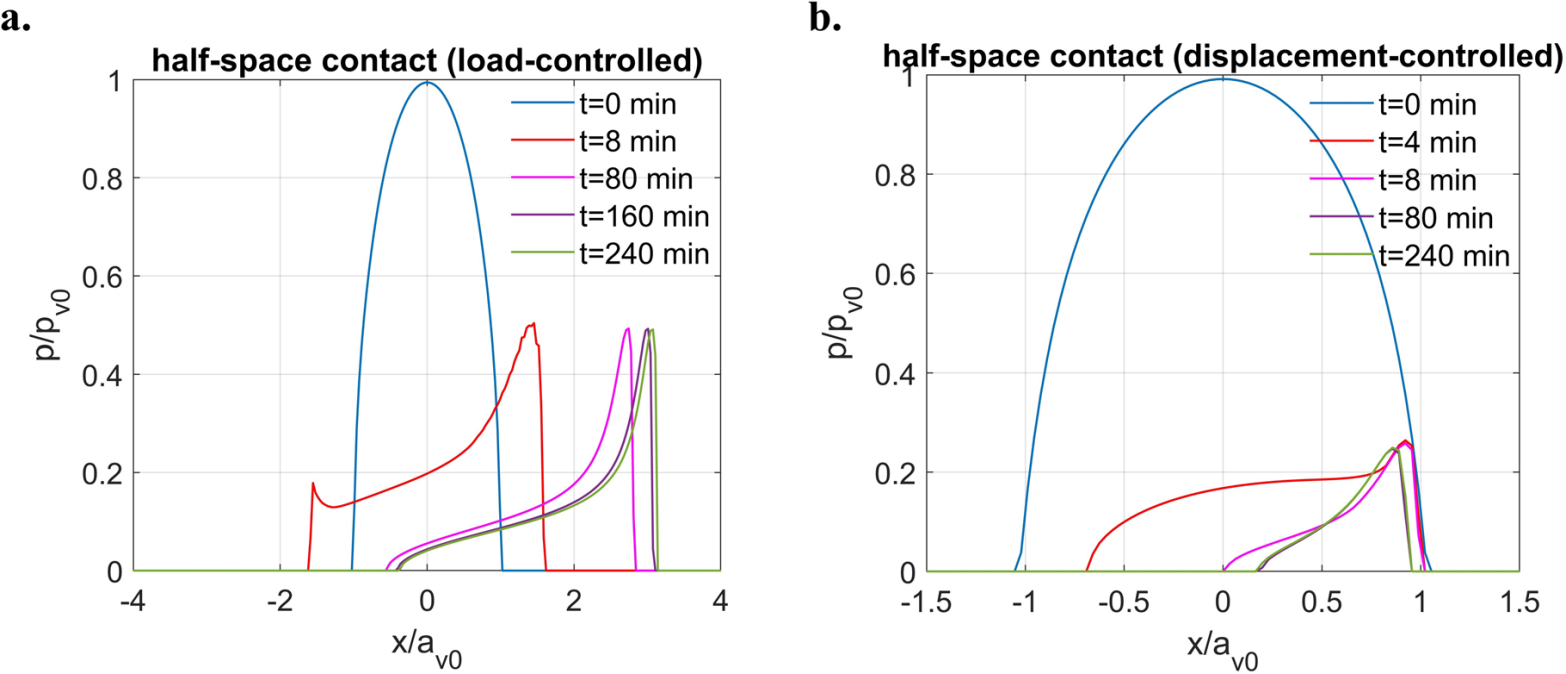
Contact solutions to frictionless sliding problems of ZDDP-derived tribofilm in the form of half-space under a fixed sliding speed $\left( {v = 0.4028\, \upmu {\text{m}}/s} \right)$ with different contact inputs: **a** $W=60 \ \text{N}$ and **b** $\delta_{z} = 36.1380 \,\upmu {\text{m}}$

To investigate the effects of tribofilm in sliding contact, the simulation starts with the case when the tribofilm exists as a half-space while the carbon steel sphere slides against it for reference. The sliding speed is assumed to be constant during the contact. To facilitate the sliding simulation, the speed is set to be $0.4028 \ \upmu {\text{m}}/{\text{s}}$ (extremely small speed in practice) as it allows a quick computation while providing a typical fluid-like contact response in the long run. As shown in Fig. 13a, under a constant normal load $(W=60 \ \text{N})$, the expanded contacting area keeps shifting in the sliding direction with time. As a result, the pressure profile is skewed significantly such that pressure spikes are observed on the leading edge of the contacting area for the plotted time points. On the other hand, when a constant surface displacement $\left( {\delta_{z} = 36.138 \ \upmu {\text{m}}} \right)$ is specified in the normal direction, as illustrated in Fig. 13b, apart from the phenomenon that the pressure relaxes with time, the rear part of the contacting area is found to keep decreasing with time. A sharp pressure spike can also be observed on the leading edge of the contacting area in this case.

When the tribofilm performs as a layer with an ultra-low thickness $(h=150 \ \text{nm})$, the combination of the tribofilm and substrate may still exhibit certain viscoelastic responses. In the following simulations, different load-controlled-based tests were conducted to investigate the effects of tribofilm under different operating conditions, including loads and sliding speeds.

Considering that the problem being simulated is on the length scale of micrometers (${a}_{0}$ ranges from 40 to 160 μm), an extremely large simulation domain along with a substantial number of time steps are needed if a speed on the scale of millimeters per second needs to be specified, making the simulation computationally intensive. For example, obtaining the simulation result for the high-speed tests ($v=1.611 \ \text{mm}/\text{s}$) under low or high loading conditions, as shown in Fig. 15e and f respectively in Appendix B, requires approximately nine hours of computation on a desktop computer with 6 cores. Instead of providing a quantitative analysis on the behaviour of ZDDP tribofilm under an exact operating environment, the study aims to obtain a qualitative trend regarding the relationship between the sliding speed and viscoelastic response of tribofilm. To achieve decent computational efficiency and accuracy, the high speeds that are specified in the following simulations are still relatively low. As some of the tested cases exhibit similar features, limited results are shown and discussed below with more test results shown in Appendix B.

**Fig. 14:**
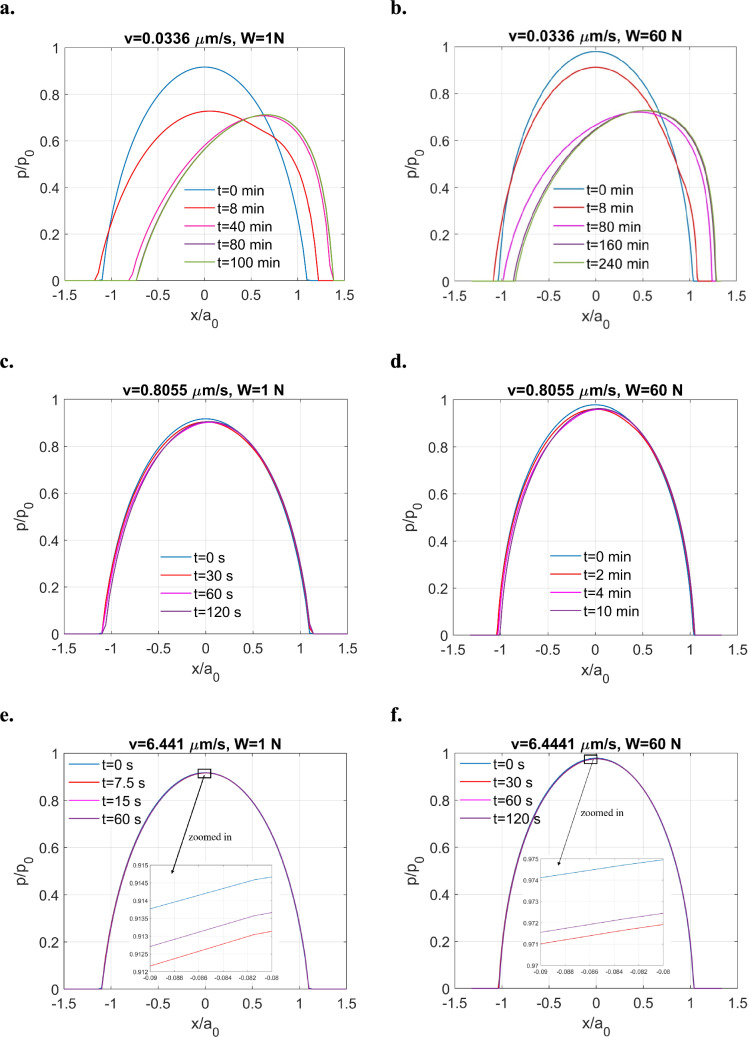
Contact solutions for frictionless sliding of ZDDP-derived tribofilm layer under varying loading conditions and sliding speeds: **a** $v = 0.0336\, \upmu {\text{m}}/{\text{s}}, W = 1 \ {\text{N}}$, **b** $v = 0.0336\, \upmu {\text{m}}/{\text{s}},W = 60 \ {\text{N,}}$ **c** ${v = 0.8055 \,\upmu {\text{m}}/{\text{s}},W = 1 \ {\text{N}}}$, **d** $v = 0.8055\, \upmu {\text{m}}/{\text{s}},W = 60 \ {\text{N}}$, **e** $v = 6.441 \,\upmu {\text{m}}/{\text{s}}, W = 1 \ {\text{N}}$ and **f** $v = 6.441 \,\upmu {\text{m}}/{\text{s}}, W = 60 \ {\text{N}}$

**Fig. 15:**
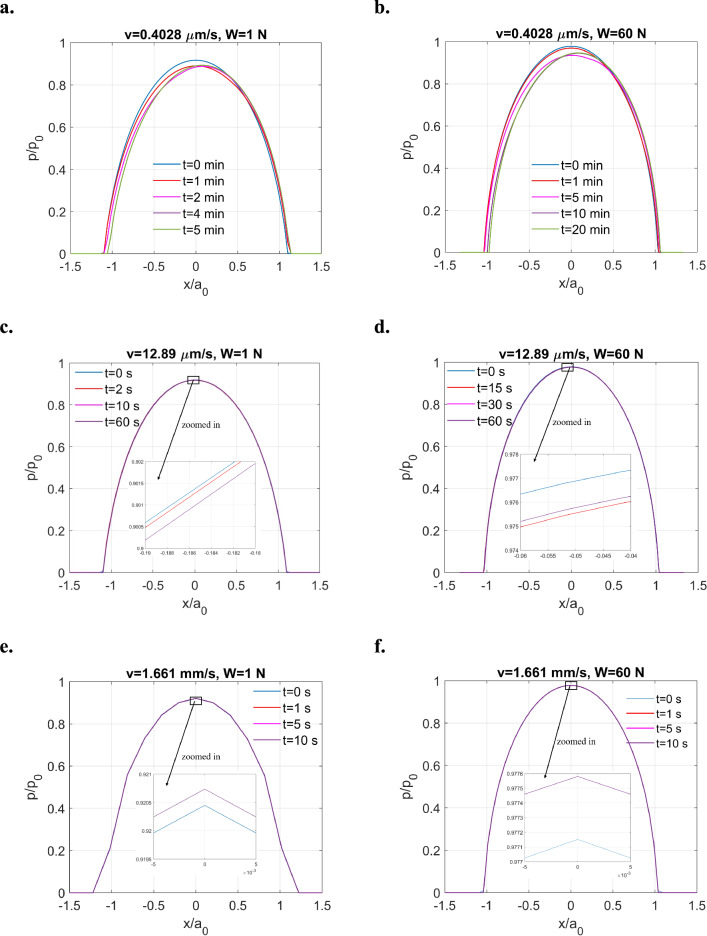
Extra sliding solutions for layered tribofilm at varying inputs: **a** $v = 0.4028 \,\upmu {\text{m}}/{\text{s}}, W = 1 \ {\text{N}}$, **b** $v = 0.4028\, \upmu {\text{m}}/{\text{s}}, W = 60 \ {\text{N}}$, **c** $v = 12.89 \,\upmu {\text{m}}/{\text{s}},W = 1 \ {\text{N}}$*,* **d** $v = 12.89\, \upmu {\text{m}}/{\text{s}},W = 60 \ {\text{N}}$*,* **e** $v = 1.661 \ {\text{mm}}/{\text{s}}, W = 1 \ {\text{N}}$ and **f** $v=1.661 \ \text{mm}/\text{s}, W=60 \ \text{N}$

At an extremely low sliding speed $(v = 0.0336 \,\upmu {\text{m}}/{\text{s}})$, the contact solutions exhibit pronounced viscoelastic characteristics under both loading conditions, such as pressure relaxation and shifting contacting areas shown in Fig. 14a and b. However, these effects are less significant compared to the half-space case, where distinct pressure spikes are observed at a higher sliding speed. Notably, when the sliding speed in the layered test is increased to the same value specified in the half-space test $\left( {v = 0.4028 \,\upmu {\text{m}}/{\text{s}}} \right)$, the viscoelastic effect of the tribofilm would be less pronounced, as illustrated in Fig. 15a and b in Appendix B.

When the sliding speed increases to $v = 0.8055 \,\upmu {\text{m}}/{\text{s}},$ the viscoelastic responses of the two loading cases become much less significant, where only micro changes of pressure distributions can be observed as shown in Fig. 14c and d while the contacting area no longer shifts significantly with time.

After further increasing the sliding speed to $v = 6.441 \,\upmu {\text{m}}/{\text{s,}}$ time-dependent changes of the contact solutions can hardly be observed for both loading conditions, where micro changes of pressure distributions with time can only be observed in the zoomed-in views shown in Fig. 14e and f. It is of note that it always takes longer time for the high loading case $(W=60 \ \text{N})$ to reach a steady state for all three tests conducted at different sliding speeds.

The simulation outcome highlights the dual role of the tribofilm. On one hand, in ultra-low-speed sliding or indentation cases, the viscoelasticity of tribofilms has pronounced effects on contact solutions. It gradually reduces the contact pressure over time for metal-to-metal contact. The reduced pressure can lead to the decline of subsurface stress and subsequently contribute to mitigation of surface wear. Additionally, the damping mechanism provided by the viscosity of the tribofilm can absorb a portion of the energy that would otherwise contribute to material wear. Dorgham, et al. [25, 27] conducted nanoscale experiments with sliding speed varying on the scale of micrometer per second, effectively capturing and validating the role of viscoelasticity in enhancing wear resistance.

On the other hand, these potential benefits are speed-dependent (scale-dependent) and may diminish when operating conditions change. As mentioned before, the sliding speed considered in this study is relatively low, especially when compared to the typical operating conditions where ZDDP additives are used. For example, in cylinder liner piston ring assemblies, the average sliding speed is reported to range from 0.08 to 0.32 m/s [68], and similar speeds ranging from millimeters to meters per second are employed in tribofilm formation experiments [69, 70]. At these high sliding speeds, the viscoelastic response of the layered contact becomes negligible. Tribofilms primarily behave as soft elastic layers, with pressure reduction occurring primarily during the initial contact and stabilizing shortly thereafter. Consequently, the potential impact of viscoelasticity on wear reduction shall be less significant.

## Conclusion

By converting frequency response functions to influence coefficients, the model for viscoelastic layered contact is developed on the basis of the half-space contact model. The model is then extended to investigate the viscoelastic behaviour of a ZDDP-derived tribofilm, which is characterized by a Burgers material model.

The contact response of tribofilm is found to be extremely fluid-like when it exists as a half-space body. When the tribofilm is bonded to an elastic solid, for example a carbon steel substrate being considered in this study, viscoelastic contact responses, including creep and stress relaxation can be observed, while the extent of which is not as significant as that in the half-space case. In the layered indentation case, the contact exhibits more remarkable viscoelastic response (creep or stress relaxation) under low-loading conditions. Additionally, it is found that when a constant surface displacement, which is not significantly lower than the layer thickness, is applied, the contacting area increases gradually with time due to the flow of the tribofilm being bounded to the elastic substrate. When it comes to layered sliding contact problems, significant time-dependent solutions are only observed under extremely low sliding speeds $(v < 1 \,\upmu {\text{m}}/{\text{s}})$, where the high loading cases exhibit more significant viscoelastic effects. On the other hand, negligible changes of pressure are observed under moderate sliding speeds ranging from millimeters to meters per second.

Given that ZDDP additives are typically applied in scenarios involving sliding or mixed sliding-rolling motions, which promote the formation of the protective tribofilm on metal surfaces through tribochemistry, the results suggest that the contact analysis of tribofilm can be simplified to a soft elastic layered contact problem depending on the sliding velocity where the ZDDP additive is applied.

Therefore, the effectiveness of ZDDP-derived tribofilms as protective layers is not only governed by their inherent material properties but also by the operating conditions. At higher speeds, where viscoelastic effects are negligible, the performance of tribofilm is dominated by its instant elastic response. Conversely, under extremely low-speed or static conditions (e.g. the work of Dorgham, et al. [25, 27]), viscoelasticity can emerge as a critical factor influencing pressure distribution, and wear mitigation. By integrating numerical, experimental and practical perspectives, a clear distinction is established between low-speed, viscoelastic-dominated responses, and high-speed, elastic-dominated tribofilm behaviours. This insight bridges the gap between the nanoscale findings of Dorgham on the viscoelastic nature of tribofilms and large-scale experimental and practical observations, where tribofilms predominantly act as soft and elastic layers [2, 17].

Based on this, how the interplay of loading conditions, film thickness, and speed variations affect the transition between viscoelastic and elastic-dominated behaviour for the tribofilms derived from different sources could be explored in the future work to provide deeper insights into optimizing additive performance for specific applications. Additionally, exploring the transient effects of tribofilm formation and removal can enhance the understanding of contact mechanics and provide more accurate solutions for tribofilm-affected systems.

## Supplementary Information

Below is the link to the electronic supplementary material.

Supplementary file1 (DOCX 928 KB)

## Data Availability

No datasets were generated or analysed during the current study.

## Appendix A

Frequency response function of surface displacements in elastic layered half-space due to $p$:

$${\widetilde{\widetilde{C}}}_{p}^{{u}_{z}}(m,n,0)=\frac{1}{2{G}_{c}}[-\alpha \left({A}^{\left(1\right)}-{\overline{A} }^{\left(1\right)}\right)-(3-4{\nu }_{1})({C}^{\left(1\right)}+{\overline{C} }^{(1)})],$$

or

$${\widetilde{\widetilde{C}}}_{p}^{{u}_{z}}\left(m,n,0\right)=\frac{{\nu }_{c}-1}{{G}_{c}}\left(1+4\alpha hk\theta -\lambda k{\theta }^{2}\right)\alpha R,$$

$${\widetilde{\widetilde{C}}}_{p}^{{u}_{x}}\left(m,n,0\right)=\frac{1}{2{G}_{c}}[im({A}^{\left(1\right)}+{\overline{A} }^{(1)})],$$

$${\widetilde{\widetilde{C}}}_{p}^{{u}_{y}}\left(m,n,0\right)=\frac{1}{2{G}_{c}}[in({A}^{\left(1\right)}+{\overline{A} }^{(1)})],$$

$$\alpha =\sqrt{{m}^{2}+{n}^{2}} , \theta ={e}^{-2\alpha h}, G=\frac{{G}_{c}}{{G}_{s}}, k=\frac{G-1}{G+3-4{\nu }_{c}},$$

$$\lambda =1-\frac{4\left(1-{\nu }_{c}\right)}{1+G\left(3-4{\nu }_{s}\right)} , R=-\frac{1}{\left[1-\left(\lambda +k+4k{\alpha }^{2}{h}^{2}\right)\theta +\lambda k{\theta }^{2}\right]{\alpha }^{2}},$$

$$A^{\left( 1 \right)} = R\left\{ { - \left( {1 - 2\nu_{c} } \right)\left[ {1 - \left( {1 - 2\alpha h} \right)k\theta } \right] + \frac{1}{2}\left( {k - \lambda - 4k\alpha^{2} h^{2} } \right)\theta } \right\},$$

$$\overline{A}^{\left( 1 \right)} = R\theta \left\{ {\left( {1 - 2\nu_{c} } \right)k\left( {1 + 2\alpha h - \lambda \theta } \right) + \frac{1}{2}\left( {k - \lambda - 4k\alpha^{2} h^{2} } \right)} \right\},$$

$$C^{\left( 1 \right)} = \left[ {1 - \left( {1 - 2\alpha h} \right)k\theta } \right]\alpha R,$$

$${\overline{C} }^{(1)}=\left(1+2\alpha h-\lambda \theta \right)k\theta \alpha R.$$

Frequency response function of surface displacements in elastic layered half-space due to ${q}_{x}$:

$${\widetilde{\widetilde{C}}}_{{q}_{x}}^{{u}_{z}}\left(m,n,0\right)=\frac{1}{2{G}_{c}}[-\alpha \left({A}^{\left(1\right)}-{\overline{A} }^{\left(1\right)}\right)-(3-4{\nu }_{1})({C}^{\left(1\right)}+{\overline{C} }^{(1)})],$$

$${\widetilde{\widetilde{C}}}_{{q}_{x}}^{{u}_{x}}\left(m,n,0\right)=\frac{1}{2{G}_{c}}[im\left({A}^{\left(1\right)}+{\overline{A} }^{\left(1\right)}\right)-4\left(1-{\nu }_{1}\right)\left({B}^{\left(1\right)}+{\overline{B} }^{\left(1\right)}\right)-m({B}_{,m}^{\left(1\right)}+{{\overline{B} }_{,m}}^{\left(1\right)})],$$

$${\widetilde{\widetilde{C}}}_{{q}_{x}}^{{u}_{y}}\left(m,n,0\right)=\frac{1}{2{G}_{c}}[in\left({A}^{\left(1\right)}+{\overline{A} }^{\left(1\right)}\right)-n({B}_{,m}^{\left(1\right)}+{{\overline{B} }_{,m}}^{\left(1\right)})],$$

$${B}_{,m}=\partial B/{\partial }_{m},$$

$$\alpha =\sqrt{{m}^{2}+{n}^{2}} , \theta ={e}^{-2\alpha h}, G=\frac{{G}_{c}}{{G}_{s}}, k=\frac{G-1}{G+3-4{\nu }_{c}}, \lambda =1-\frac{4\left(1-{\nu }_{c}\right)}{1+G\left(3-4{\nu }_{s}\right)},$$

$$S_{0} = \left[ {G + \left( {3 - 4\nu_{c} } \right)} \right]\left( {1 - k\theta } \right),$$

$$\overline{B}^{\left( 1 \right)} = - \frac{{\left( {G - 1} \right)\theta }}{{\left( {1 + G} \right) + \left( {1 - G} \right)\theta }} \cdot \frac{1}{{2\alpha \left( {1 - \nu_{c} } \right)}},$$

$$B^{\left( 1 \right)} = \overline{B}^{\left( 1 \right)} - \frac{1}{{2\alpha \left( {1 - \nu_{c} } \right)}},$$

$$B^{\left( 2 \right)} = - \frac{{2\left( {1 - \nu_{c} } \right)}}{{1 - \nu_{s} }} \cdot \frac{\sqrt \theta }{{\left( {1 + G} \right) + \left( {1 - G} \right)\theta }} \cdot \frac{1}{{2\alpha \left( {1 - \nu_{c} } \right)}},$$

$$C^{\left( 1 \right)} = \frac{{\left( {1 - \lambda } \right)S_{0} R_{c} }}{{\left\{ {4\left( {1 - \nu_{c} } \right)\left( {G + 3 - 4\nu_{c} } \right)\left[ {1 - \left( {\lambda + k + 4k\alpha^{2} h^{2} } \right)\theta + \lambda k\theta^{2} } \right]} \right\}}},$$

$$\overline{C}^{\left( 1 \right)} = \frac{{\left[ {2\left( {G - 1} \right)\alpha h\theta C^{\left( 1 \right)} + \theta R_{a} } \right]}}{{S_{0} }},$$

$$A^{\left( 1 \right)} = \frac{{\left[ { - \left( {3 - 4\nu_{c} } \right)C^{\left( 1 \right)} + \overline{C}^{\left( 1 \right)} + \alpha \left( {R_{1} + R_{2} } \right)} \right]}}{2\alpha },$$

$$\overline{A}^{\left( 1 \right)} = \frac{{\left\{ {\left( {1 - \lambda } \right)\theta C^{1} + \left[ {\left( {3 - 4\nu_{c} } \right)\left( {1 - \theta } \right) - 2\alpha h} \right]\overline{C}^{\left( 1 \right)} + \theta R_{d} } \right\}}}{{\left[ {2\alpha \left( {1 - \theta } \right)} \right]}},$$

$$C^{\left( 2 \right)} = \{ \left[ {4\left( {1 - \nu_{c} } \right)\left( {1 - \lambda } \right)\sqrt \theta } \right]C^{\left( 1 \right)} + \left( {1 - \lambda } \right)\alpha \sqrt \theta (R_{3} - R_{4} + R_{5} - R_{6} )\} /\left[ {4\left( {1 - \nu_{c} } \right)} \right],$$

$$A^{\left( 2 \right)} = \left\{ {2\alpha h\sqrt \theta \left[ {S_{0} - \left( {G - 1} \right)\left( {1 - \theta } \right)} \right]C^{\left( 1 \right)} - \left[ {\left( {3 - 4\nu_{s} } \right)S_{0} } \right]C^{\left( 2 \right)} + \alpha \sqrt \theta S_{0} \left( {R_{1} + R_{2} - R_{3} - R_{4} } \right) - \sqrt \theta \left( {1 - \theta } \right)R_{a} } \right\}/\left( {2\alpha S_{0} } \right),$$

$$-{\alpha }^{2}{R}_{1}=im\left({B}^{\left(1\right)}-{\overline{B} }^{\left(1\right)}\right)+i{\alpha }^{2}\left({B}_{m}^{\left(1\right)}-{\overline{B} }_{m}^{\left(1\right)}\right),$$

$$-{\alpha }^{2}{R}_{2}=2im\left(1-{\nu }_{c}\right)\left({B}^{\left(1\right)}+{\overline{B} }^{\left(1\right)}\right)+i{\alpha }^{2}\left({B}_{m}^{\left(1\right)}+{\overline{B} }_{m}^{\left(1\right)}\right),$$

$$-{\alpha }^{2}{R}_{3}=i\left(m-m\alpha h\right){B}^{\left(1\right)}-i\left(m+m\alpha h\right){\theta }^{-1}{\overline{B} }^{\left(1\right)}-im{\theta }^{-\frac{1}{2}}{B}^{\left(2\right)}+i{\alpha }^{2}{B}_{,m}^{\left(1\right)}-i{\alpha }^{2}{\theta }^{-1}{\overline{B} }_{,m}^{\left(1\right)}-i{\alpha }^{2}{\theta }^{-\frac{1}{2}}{\overline{B} }_{,m}^{\left(2\right)},$$

$$-{\alpha }^{2}{R}_{4}=\left[2i\left(1-{\nu }_{1}\right)m-im\alpha h\right]{B}^{\left(1\right)}+\left[2i\left(1-{\nu }_{1}\right)m+im\alpha h\right]{\theta }^{-1}{\overline{B} }^{\left(1\right)}-\left[2i\left(1-{\nu }_{2}\right)m\right]{\theta }^{-\frac{1}{2}}{B}^{\left(2\right)}+i{\alpha }^{2}{B}_{,m}^{\left(1\right)}+i{\alpha }^{2}{\theta }^{-1}{\overline{B} }_{,m}^{\left(1\right)}-i{\alpha }^{2}{\theta }^{-\frac{1}{2}}{\overline{B} }_{,m}^{\left(2\right)},$$

$$-{\alpha }^{2}{R}_{5}=-im\alpha h{B}^{\left(1\right)}+im\alpha h{\theta }^{-1}{\overline{B} }^{\left(1\right)}+i{\alpha }^{2}{B}_{,m}^{\left(1\right)}+i{\alpha }^{2}{\theta }^{-1}{\overline{B} }_{,m}^{\left(1\right)}-iG{\alpha }^{2}{\theta }^{-\frac{1}{2}}{\overline{B} }_{,m}^{\left(2\right)},$$

$$-{\alpha }^{2}{R}_{6}=i\left(m-m\alpha h\right){B}^{\left(1\right)}-i\left(m+m\alpha h\right){\theta }^{-1}{\overline{B} }^{\left(1\right)}-iGm{\theta }^{-\frac{1}{2}}{B}^{\left(2\right)}+i{\alpha }^{2} {B}_{,m}^{\left(1\right)}-i{\alpha }^{2}{\theta }^{-1}{\overline{B} }_{,m}^{\left(1\right)}-iG{\alpha }^{2}{\theta }^{-\frac{1}{2}}{\overline{B} }_{,m}^{\left(2\right)},$$

$${R}_{a}=\left(G-1\right)\alpha \left({R}_{1}+{R}_{2}\right)-G\alpha \left({R}_{3}+{R}_{4}\right)+\alpha \left({R}_{5}+{R}_{6}\right),$$

$${R}_{b}=\alpha \left({R}_{2}-{R}_{1}\right)+\alpha \theta \left({R}_{3}-{R}_{4}\right),$$

$${R}_{c}=\frac{4\left(1-{\nu }_{1}\right)}{1-\lambda }\left(\frac{2\alpha h\theta }{{S}_{0}}{R}_{a}+{R}_{b}\right)-\alpha \theta \left({R}_{3}-{R}_{4}+{R}_{5}-{R}_{6}\right),$$

$${R}_{d}=\alpha \left({R}_{1}-{R}_{2}-{R}_{3}+{R}_{4}\right)+\frac{\left(1-\lambda \right)\alpha }{4\left(1-{\nu }_{1}\right)}\left({R}_{3}-{R}_{4}+{R}_{5}-{R}_{6}\right).$$

Frequency response function of surface displacements in elastic layered half-space due to ${q}_{y}$ can be determined from symmetrical characters:

$${\widetilde{\widetilde{{C}}}_{{q}_{y}}^{{u}_{x}}}\left(m,n,0\right)={\widetilde{\widetilde{{C}}}_{{q}_{x}}^{{u}_{y}}}\left(n,m,0\right), {\widetilde{\widetilde{{C}}}_{{q}_{y}}^{{u}_{y}}}\left(m,n,0\right)={\widetilde{\widetilde{{C}}}_{{q}_{x}}^{{u}_{x}}}\left(n,m,0\right), {\widetilde{\widetilde{{C}}}_{{q}_{y}}^{{u}_{z}}}\left(m,n,0\right)=-{\widetilde{\widetilde{{C}}}_{p}^{{u}_{x}}}\left(n,m,0\right)$$

## Appendix B

See Fig. 15.

Notably, for the high-speed case ($v=1.661 \ \text{mm}/\text{s}$), the sliding contact quickly reaches the steady state, showing consistent results at $t=1 \ \text{s}$, $t=5 \ \text{s}$, and $t=10 \ \text{s}$.
