# Supplementary material for: Time-Dependent Contact Behaviour of ZDDP-Derived Tribofilms: A Viscoelastic Layered Model Approach
Source: Tribol Lett. 2025 May 3;73(2):69. doi: 10.1007/s11249-025-01990-5 (PMC12049326; doi:10.1007/s11249-025-01990-5)
Supplement: Supplementary file 1 — Supplementary file1 (DOCX 928 KB) [file 11249_2025_1990_MOESM1_ESM.docx]

Time-Dependent Contact Behaviour of ZDDP-Derived Tribofilms: A Viscoelastic Layered Model Approach

Dongze Wang^1*^, Ali Ghanbarzadeh^1^, Nan Xu^1^, Qingyang Liu^2^, Gregory de Boer^1^

^1^ School of Mechanical Engineering, University of Leeds, Leeds, LS2 9JT, UK

^2^ Qatar Environment and Energy Research Institute, Doha, Qatar

Supplementary Materials

Sensitivity of Simulation Outcome to Computational Parameters

The numerical study aims to check if the tribofilm contact results are insensitive to specified computational parameters. Two representative cases, including an indentation contact problem and a frictionless sliding contact problem, were investigated.

## Indentation Contact Problem

The following three different spatial discretizations shown in Table S 1 were specified to simulate the same indentation contact problem, where a normal load of $60 N$ is specified for the tribofilm layered contact problem $(h=150 \mathrm{nm})$.

Table S 1. Grid parameters specified for the simulation of indentation contact problems

| Test No. | Size of computational domain | Number of nodes | Pixel width ($\mu m$) |
| --- | --- | --- | --- |
| 1 | $0.2a_{max}\times0.2a_{max}$ | $256\times256$ | 2.9455 |
| 2 | $0.2a_{max}\times0.2a_{max}$ | $128\times128$ | 5.9142 |
| 3 | $0.8a_{max}\times0.8a_{max}$ | $256\times256$ | 11.7820 |

The simulation results of the three tests are shown in Fig S 1, where the contact solutions of the three tests agree closely. Minor differences can be observed from the two zoomed-in views, the magnitude of which becomes smaller when the contact reaches the steady state ($t=120 \min$). The coarsest mesh (Test 3) produces slightly less smooth pressure gradients, but without altering the overall trends.


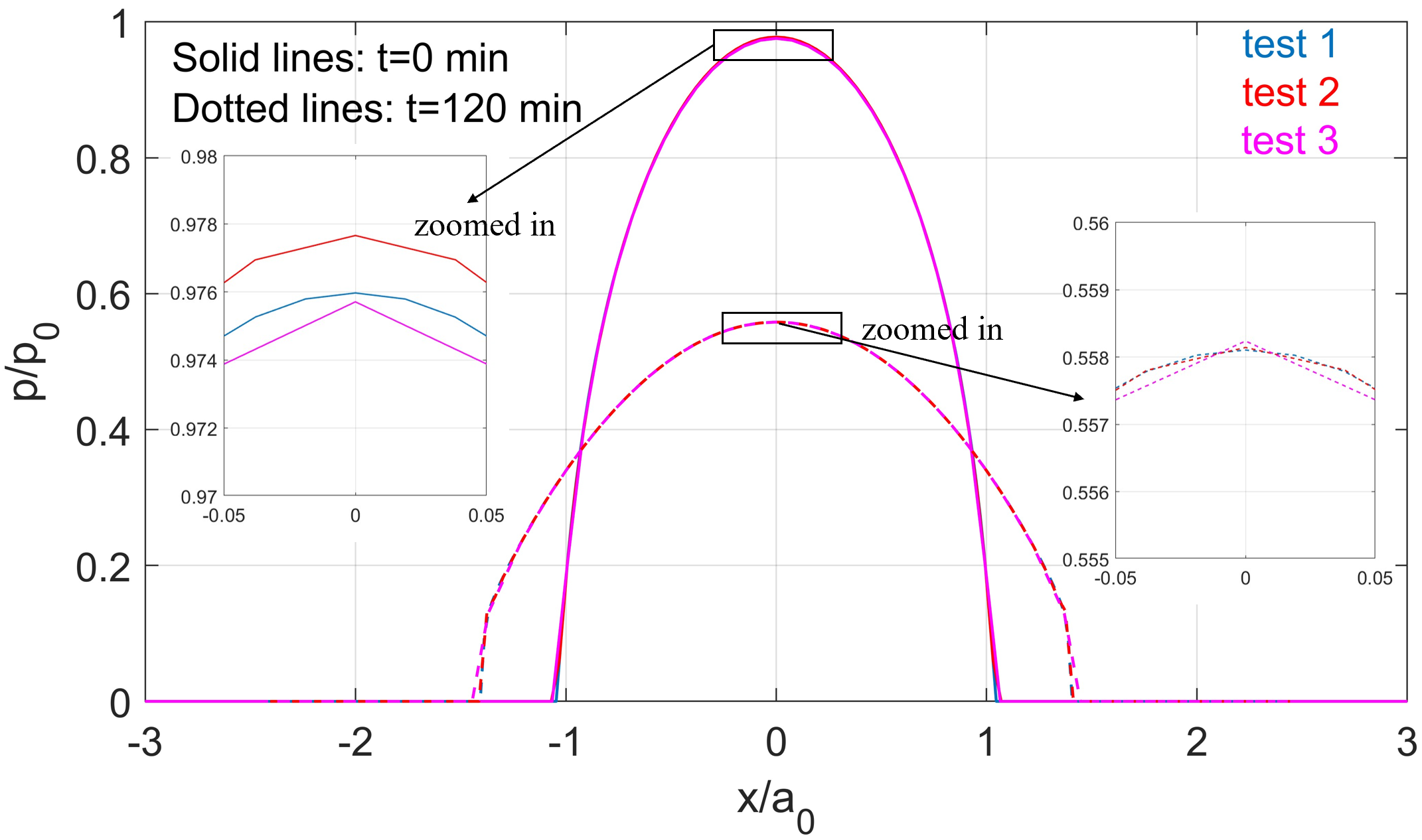


Fig S 1. Simulation outcome of indentation problems at different times $\boldsymbol{t=0}\mathbf{min}$ and $\boldsymbol{t=120}\mathbf{min}$ with different computational parameters

## Sliding Contact Problem

For the frictionless sliding case ($W=1 N$, $h=150 \mathrm{nm}$ and $v=0.0336 \mu m/s$), both spatial and temporal discretisation were varied, details of which are given in Table S 2 and Table S 3 respectively.

Table S 2. Grid parameters specified for the simulation of sliding contact problems

| Test No. | Size of computational domain | Number of nodes | Pixel width ($\mu m$) |
| --- | --- | --- | --- |
| 1 | $0.6875a_{0}^{*}\times0.6875a_{0}^{*}$ | $512\times512$ | $0.4020$ |
| 2 | $1.375a_{0}^{*}\times1.375a_{0}^{*}$ | $512\times512$ | $0.8039$ |
| 3 | $1.375a_{0}^{*}\times1.375a_{0}^{*}$ | $256\times256$ | $1.6110$ |

Table S 3 Temporal parameters specified for the simulation of sliding contact problems

| Test No. | Total simulation time (min) | Number of time steps | Time interval (s) |
| --- | --- | --- | --- |
| 1 | $100$ | $501$ | $12$ |
| 2 | $100$ | $251$ | $24$ |
| 3 | $100$ | $126$ | $48$ |

The simulation outcomes of the three tests are shown in Fig S 2. When the viscoelastic sliding contact initializes $(t=0 \min)$, all three tests yield nearly identical pressure distributions, where the results of test 1 and test 2 overlap. Test 3 exhibits negligible difference as shown in the zoomed-in view. When the sliding contact tends to become steady ($t=100 \min$), minor quantitative differences appear, owing to the combined effects of coarser spatial resolution and large time intervals. However, all simulations exhibit the same qualitative behaviour.


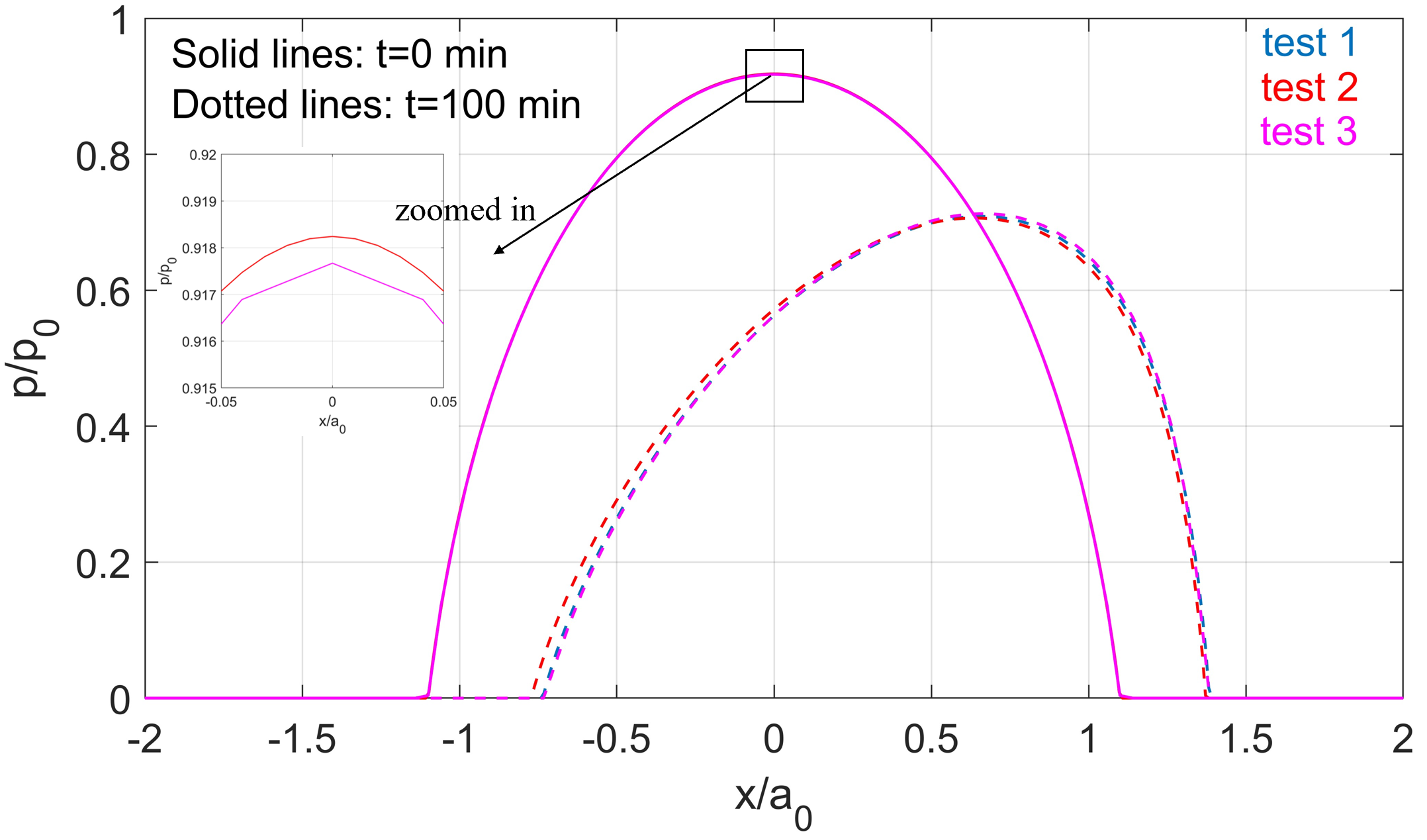


Fig S 2 Simulation outcome of sliding problems at different times $\boldsymbol{t=0}\mathbf{min}$ and $\boldsymbol{t=100}\mathbf{min}$ with different computational parameters, the solution of test 1 and test 2 coincide at $\boldsymbol{t=0}\mathbf{min}$

Overall, mesh refinement beyond the parameters used in our main simulations (conditions specified in test 3 for the two investigated cases) does not significantly change the pressure distribution or qualitative trends. Only minor gradient discontinuities emerge for very coarse discretisation, which confirms that the chosen computational parameters yield robust solutions, though the accuracy can be improved at the expense of computational efficiency.
